# Transcriptomic analysis of differential host gene expression upon uptake of symbionts: a case study with *Symbiodinium* and the major bioeroding sponge *Cliona varians*

**DOI:** 10.1186/1471-2164-15-376

**Published:** 2014-05-16

**Authors:** Ana Riesgo, Kristin Peterson, Crystal Richardson, Tyler Heist, Brian Strehlow, Mark McCauley, Carlos Cotman, Malcolm Hill, April Hill

**Affiliations:** Department of Organismic and Evolutionary Biology, Harvard University, Barcelona, Spain; Department of Animal Biology, Universitat de Barcelona, Barcelona, Spain; Department of Biology, University of Richmond, Richmond, VA USA; Department of Molecular Physiology and Biophysics, Vanderbilt School of Medicine, Nashville, TN USA; Department of Cell Biology, University of Virginia, Charlottesville, VA USA; University of Western Australia, Australian Institute of Marine Science, Perth, Australia; Department of Biology, University of Mississippi, University, MS USA

**Keywords:** Symbiosis, Genetic integration, Porifera, Transcriptome, Zooxanthellae

## Abstract

**Background:**

We have a limited understanding of genomic interactions that occur among partners for many symbioses. One of the most important symbioses in tropical reef habitats involves *Symbiodinium*. Most work examining *Symbiodinium*-host interactions involves cnidarian partners. To fully and broadly understand the conditions that permit *Symbiodinium* to procure intracellular residency, we must explore hosts from different taxa to help uncover universal cellular and genetic strategies for invading and persisting in host cells. Here, we present data from gene expression analyses involving the bioeroding sponge *Cliona varians* that harbors Clade G *Symbiodinium*.

**Results:**

Patterns of differential gene expression from distinct symbiont states (“normal”, “reinfected”, and “aposymbiotic”) of the sponge host are presented based on two comparative approaches (transcriptome sequencing and suppressive subtractive hybridization (SSH)). Transcriptomic profiles were different when reinfected tissue was compared to normal and aposymbiotic tissue. We characterized a set of 40 genes drawn from a pool of differentially expressed genes in “reinfected” tissue compared to “aposymbiotic” tissue via SSH. As proof of concept, we determined whether some of the differentially expressed genes identified above could be monitored in sponges grown under ecologically realistic field conditions. We allowed aposymbiotic sponge tissue to become re-populated by natural pools of *Symbiodinium* in shallow water flats in the Florida Keys, and we analyzed gene expression profiles for two genes found to be increased in expression in “reinfected” tissue in both the transcriptome and via SSH. These experiments highlighted the experimental tractability of *C. varians* to explore with precision the genetic events that occur upon establishment of the symbiosis. We briefly discuss lab- and field-based experimental approaches that promise to offer insights into the co-opted genetic networks that may modulate uptake and regulation of *Symbiondinium* populations *in hospite*.

**Conclusions:**

This work provides a sponge transcriptome, and a database of putative genes and genetic pathways that may be involved in *Symbiodinium* interactions. The relative patterns of gene expression observed in these experiments will need to be evaluated on a gene-by-gene basis in controlled and natural re-infection experiments. We argue that sponges offer particularly useful characteristics for discerning essential dimensions of the *Symbiodinium* niche.

**Electronic supplementary material:**

The online version of this article (doi:10.1186/1471-2164-15-376) contains supplementary material, which is available to authorized users.

## Background

It is a truism that most if not all species on the planet serve as habitat for one or more microbial symbiont [1]. These associations can have ecological outcomes that are beneficial (e.g., mutualisms) or deleterious (e.g., parasitisms), and as such are among the most important biological interactions on the planet given that they affect everything from general ecosystem health to human disease. However, our understanding of many major facets of the evolutionary and ecological interactions that occur among partners is limited. New molecular tools and a growing genomic perspective are offering the ability to discern nuanced aspects of host:symbiont interactions while identifying genes and pathways involved in regulating host:symbiont relationships [2]. Here, we employed transcriptomic approaches to elucidate the molecular genetic machinery in operation during re-establishment of an intracellular symbiosis.

The structure and function of coral reefs depends upon trophic interactions that occur between a dinoflagellate symbiont belonging to the diverse lineage referred to as *Symbiodinium* (Alveolata: Dinoflagellata: Suessioids) and a variety of invertebrate and protistan hosts [3–6]. The algal partners, known colloquially as zooxanthellae, have long been known to be of vital trophic importance to the host ([7–12]). We understand less about the benefits the symbionts receive from the association, though most hypotheses argue that *Symbiodinium* benefit from intracellular residency by gaining access to nutrients that are limiting outside the host (e.g. [11–13]). The partnership is arguably the most important ecological interaction that occurs in shallow tropical habitats worldwide because *Symbiodinium* spp. energetically subsidize the entire ecosystem and power calcification processes [14] that generate the topographic complexity of these systems.

Many *Symbiodinium*-based symbioses are remarkably sensitive to environmental stressors, notably elevated seawater temperatures (e.g. [15, 16]). Symbionts can be lost from the host through a process known as bleaching, which can have significant deleterious effects on the host [17]. There is growing concern among scientists about what the potential disruption of this important symbiosis means for the future of coral reefs (e.g. [18–20]). In the face of these concerns, it has become apparent that significant gaps exist in our basic comprehension of the natural dynamics of the *Symbiodinium*:host interaction, and in the degree of cellular and genetic integration among partners. Hosts can recover from mild and even massive losses of their symbiont populations, though mortality rates of the hosts increase under both scenarios, especially the latter [21]. *Symbiodinium* spp. are also capable of (in fact probably require) existence outside of the host, and *Symbiodinium* spp. have planktonic, free-living stages that occur even during non-bleaching events (e.g. [22, 23]). Currently, coral reef biologists have a limited capacity to satisfactorily explain the facultative nature of the symbiotic interaction between *Symbiodinium* and heterotrophic hosts [13]. We do not know how facile/labile the symbiotic association between *Symbiodinium* spp. and their host partners is, nor what selective landscapes are in place that favor the observed patterns of partner association. Understanding fundamental aspects of symbiont uptake, establishment of intracellular residency, and dynamics behind cellular expulsion will be essential as we attempt to manage the significant environmental changes underway on coral reefs.

As we face warming sea surface temperatures due to human-induced climate change, it has become more pressing to understand the interactions that occur among the partners at the finest molecular genetic levels so that we may better prepare for the ecological realities coral reefs will face. In the broadest terms, we lack a clear understanding of how *Symbiodinium* navigates a potential host’s cellular and molecular genetic machinery so that digestion, detection and expulsion are avoided; we also lack a clear understanding of what role the host might play in permitting intracellular residency. Recent advances in molecular and genomic approaches have enhanced our understanding of some of the regulatory operations executed between cnidarian hosts and zooxanthella symbionts (e.g. [24–33]). Molecular genetic data has failed to identify “symbiosis-specific” genes that regulate the interaction between partners, but instead has found subtle differences in expression patterns that depend on holobiont context. For example, symbiont cladal identity has been shown to play an important role in transcriptomic profiles [28]. Emphasis has now shifted toward finding the cellular pathways that are modulated such that *Symbiodinium* maintain their position within the host cell or a particular type of tissue (e.g. [27, 30, 34]).

Given that cnidarians are not the only habitable hosts for *Symbiodinium* on coral reefs (e.g. [35–38]), we stand to gain insights into nuanced aspects of the entire zooxanthella niche through analysis of non-cnidarian systems (e.g. [39]). Sponges are ecological important members of many marine ecosystems (e.g. [40, 41]), and their simple body plans affords interesting experimental opportunities [42, 43]. They belong to an ancient metazoan lineage that represents one of the earliest branches of the animal lineage [44, 45]. Sponges use flagellated choanocytes in the choanoderm to propel large volumes of water through an aquiferous system that efficiently remove bacterioplankton and dissolved organic matter while the pinacoderm mediates interaction with the environment [41].

In the work presented here, we took advantage of a suite of molecular tools to explore aspects of the intracellular symbiosis that exists between the Caribbean bioeroding sponge *Cliona varians* and its Clade G *Symbiodinium* symbionts. Sponge: zooxanthella symbioses are especially important given that *Symbiodinium* are predominantly associated with the bioeroding sponges that dissolve calcareous structures (e.g. [46]), which is a growing concern given CO_2_-driven changes in the pH of seawater [47]. Non-cnidarian systems also offer some empirical and comparative advantages over cnidarian hosts (e.g., the ability to create intracellular associations in hosts that have no evolutionary history of symbiotic associations with *Symbiodinium*[48], the ability to compare genetic expression profiles in congeneric species that differ in their ability to form symbioses with *Symbiodinium* (e.g., *C. delitrix* versus *C. varians*), and the ability to produce aposymbiotic cell aggregates (e.g. [43]) that can then be exposed to *Symbiodinium* under precisely controlled conditions). In this context, we present *C. varians* as a useful tool to better understand the *Symbiodinium* niche *sensu lato* as well as to achieve a high level of resolution of genetic regulation in sponge:*Symbiodinium* and all intracellular associations.

## Results and discussion

### Creation of “aposymbiotic” and “reinfected” tissue

**Figure 1:**
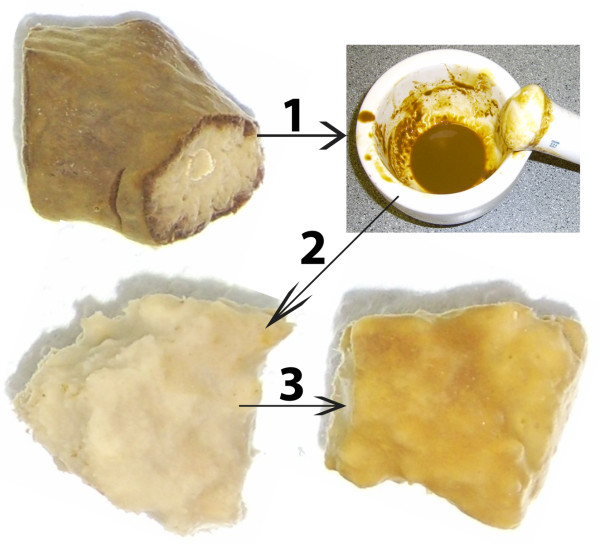
**Reinfection process involving** ***Cliona varians*** **forma** ***varians*** **.** *Symbiodinium* were released from recently collected sponges (step 1). The dark brown ring that can be seen in the cross-section of the sponge represents the pinacodermal region where *Symbiodinium* reside at high densities. The brown color comes from the symbiont populations. Aposymbiotic sponges grown in a light-tight aquarium were inoculated with *Symbiodinium* (step 2). These sponges were monitored for several days until signs of reinfection were noticeable at which point a sample was taken for subsequent molecular work (step 3).

*Cliona varians* forma *varians* associates with dense populations of Clade G *Symbiodinium*[38, 46]. These sponges can be divorced from their resident symbionts by removing the pinacodermal region of the sponge, which is the site of highest *Symbiodinium* density (Additional file 1: Figure S1). The “aposymbiotic” explants can then be reared in light-tight containers supplied with continuously flowing seawater. We were able to maintain “aposymbiotic” tissue of *C. varians* forma *varians* for months under these conditions. We discovered we were able to restore the symbiotic condition by exposing “aposymbiotic” explants to recently extracted homologous *Symbiodinium* (Figure 1). Explants from “aposymbiotic” tissue were able to take up symbionts, and after 5 days showed signs of reinfection. Thus, we were able to identify three types of sponge tissue that had different symbiont states (“normal”, “reinfected”, and “aposymbiotic” – Figure 1).

### Transcriptome characterization: de novo assembly, BLAST, and functional annotation

We sequenced transcriptomes from “normal”, “reinfected”, and “aposymbiotic” sponges. Each pool of RNA used for subsequent sequencing of the three tissue types was derived from at least three different sponge samples, but these were pooled into a single batch for each symbiont state prior to next generation sequencing. Thus, the sequences we present below come from non-replicated sequence runs (see Methods section). This caveat becomes important when interpreting the putative differences we observed. We recognize a preferable approach would be to sequence several distinct and independent samples from each symbiont state. However, this was a pilot study to determine the feasibility of using *C. varians* to study *Symbiodinium* symbioses, and used several approaches (e.g., transcriptomics, suppressive subtractive hybridization (see below)) to assess molecular genetic regulation. At the time we sequenced the transcriptomes, costs associated with sequencing multiple replicates were prohibitive. Furthermore, best practices associated with RNASeq experiments were just being developed (e.g. [49]). Nonetheless, the success we achieved in obtaining high quality sequences indicated that the database we present below will be a useful resource for the community as future studies attempt to discern significant differences observed at various stages of the establishment and maintenance of *Symbiodinium* symbioses.

The number of reads obtained from the sequencing platform HiSeq for “normal,” “aposymbiotic,” and “reinfected” treatments are shown in Table 1. The quality of the reads was highly similar across treatments: most reads with average phred score of 36, GC content from 42 to 44%, and the levels of sequence duplication varying only from 81% in “normal” to 89% “reinfected” treatment (Table 1). Before the de novo assembly, between 9 million and 34 million reads were trimmed in the separate datasets (Table 1). The number of bases assembled in contiguous sequences (contigs) was always over 21 Mb. The number of contigs ranged from 51,020 in “reinfected” to 202,907 in “normal” treatments with average contig sizes > 400 bp in all cases (Table 1). The major differences between numbers of contigs in “reinfected” vs “normal” states were due to small contigs (between 200–400 bp). The N50 for each treatment was always close to 500 bp (Table 1). The number of assembled reads and size in megabases of the dataset, as well as the number and size of contigs in all datasets, was similar to recent transcriptomic datasets published from two demosponges (*Petrosia ficiformis* and *Crella elegans*) obtained with similar methodologies ([50, 51]). Transcriptomic sequences were deposited in the NCBI Sequence Read Archive (see *Availability of Supporting Data* Section below).

**Table 1:** ***de novo*** **assembly data from the RNA-Seq experiments involving the three symbiont treatments “normal,” “reinfected,” and “aposymbiotic”**

Dataset	N reads BT	GC content (%)	Sequence duplication (%)	N reads trimmed	Avg. L AT	N contigs	N bases (Mb)	Avg. L Contigs	Max contig L	N50
**Normal**	86,048,128	44	89/88.2	21,152,025	100.6	202,907	88.0	433.7	20,547	468
**Aposymbiotic**	71,135,240	43	82/81.5	13,527,103	100.7	142,371	67.3	473.2	33,836	556
**Reinfected**	39,036,828	42	89.5/89	9,003,053	100.6	51,020	21.7	417.1	5,732	454
**Reference (pooled data)**	157,183,368	-	-	34,679,128	100.7	292,182	87.1	468.3	21,891	502

The reference category represents pooled datasets from each of the other three. Abbreviations: N, number; *BT*, before trimming; *Avg*, average; *L*, length; *AT*, after trimming; *Max*, maximum. For the sequence duplication percentages, the first number refers to the forward reads (R1) and the second to the reverse reads (R2).

**Figure 2:**
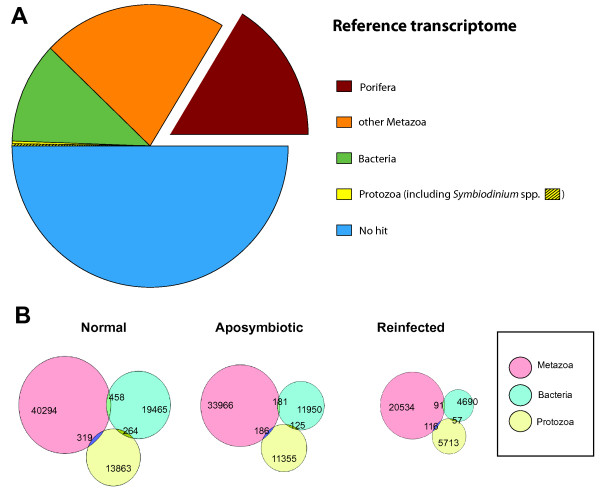
**General characteristics of** ***Cliona varians*** **transcriptomes. A**. Percentages of BLAST hits of the reference transcriptome against Porifera, other Metazoa, Bacteria, and Protozoa (including *Symbiodinium* spp.), using a combined database of Metazoa, Bacteria, and Protozoa. **B**. Hit count obtained from the independent BLAST searches for contigs of the transcriptomes of each treatment when BLAST searches were performed against only one database or two (overlap between the circles).

For non-model organisms sequenced *de novo*, it is typical that fewer than 50% of the contigs return hits against the Genbank Metazoa database when the BLAST algorithm is employed ([50–52]). For the “reference” dataset (Table 1), 61,340 sequences returned BLAST hits against Metazoa - 12% of those were specifically poriferan; another 15% were to other Metazoa (Figure 2A). Bacteria (29,908 sequences) and Protozoa (20,067 sequences - including 4,008 sequences against *Symbiodinium* spp.) were also recovered (Figure 2A). *Symbiodinium* sequences recovered included the genes *cytochrome oxidase subunit I* and *cytochrome b* (always with e-value 1e-05), which were assigned to the following taxa: *Symbiodinium goreaui* (Genbank accession: ABK5409), *S. microadriaticum* (ABK57993), *Protodinium simplex* (AEM91635), *Pelagodinium beii* (AEM91636), *Polarella glacialis* (AEM91637), *Symbiodinium* sp. cultured from *Aiptasia* sp. (AAM9012), *Symbiodinium* sp. from *Acrozoanthus australiae* (APD02933), *Symbiodinium* sp. from *Palythoa mutuki* (ACA30467), and *Symbiodinium* sp. from *Zoanthus vietnamensis* (ACA30462).

For each tissue treatment, most contig sequences with hits returned a BLAST hit against the metazoan database, followed by the bacterial database, and then the protozoan database, with very few contigs obtaining hits against more than one database (Figure 2B). The “normal” treatment obtained more BLAST hits than the other two treatments, whereas the “reinfected” treatment returned the fewest BLAST hits (Figure 2B). This difference in patterns of BLAST hits could be due to differences in sequence read numbers obtained for the different treatments (i.e., 21 M trimmed reads in control vs 9 M reads in reinfected; Additional file 2: Table S1), which could represent experimental error (i.e., technical variation). Alternatively, this pattern could point to an actual molecular genetic response to the onset of symbiosis in the form of global- or chromatin-level gene regulation (e.g. [24, 25]). For example, symbiont-induced, host-gene suppression may be a feature of the initiation of host:symbiont interactions [53]. Further data are necessary to test this hypothesis.

**Figure 3:**
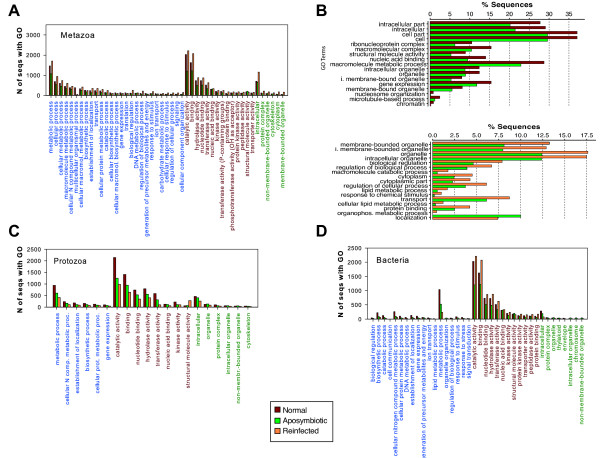
**Overview of GO term assignments in** ***Cliona varians*** **transcriptomes. A**. Selected GO term assignments in each transcriptomic dataset (“normal”, “aposymbiotic”, and “reinfected” treatments) when searched against the database Metazoa. **B**. Enriched GO terms shown in pairwise comparisons between “normal” and “aposymbiotic” treatments, and “normal” and “reinfected” treatments using the correction FDR on the Fisher’s exact test (p > 0.005) for only metazoan hits. **C-D**. Selected GO term assignments in each transcriptomic dataset (“normal”, “aposymbiotic”, and “reinfected” treatments) when searched against the databases Bacteria, and Protozoa (sub-selections of the nr database from NCBI). In the x-axis of **A**, **C-D**, the GO terms belonging to the “biological process” category are shown in blue, those belonging to “molecular function” in red, and those to “cellular component” in green.

Given the limited number of genomic and transcriptomic resources available for non-model organisms, Gene Ontology (GO) term assignment analyses return few annotated sequences, which rarely surpass 10% of the total dataset ([50–52, 54]). Of particular interest to this study are the GO term assignments showing more sequences derived from the “reinfected” treatment, which might indicate categories of genes involved in acquisition and establishment of *Symbiodinium* populations. Several of these categories were identified in our study, and included GO terms like metabolic and cellular processes, biological regulation, binding, and intracellular components (Figure 3A). A pairwise enrichment analysis of the GO terms obtained for each treatment using the Metazoa database recovered several significantly enriched terms (Figure 3B). Among the enriched GO terms in “reinfected” compared to “aposymbiotic” treatments are organelle (membrane bounded and intracellular membrane bounded), biological regulation, macromolecule catabolic process, cytoplasm, regulation of cellular process, lipid metabolic process (and cellular lipid metabolic process), response to chemical stimulus, transport, and protein binding. These categories are targets for future exploration of processes and mechanisms important in host: symbiont interactions. We found that the “reinfected” treatment often contained more bacterial and metazoan GO term assignments than the other two treatments (Figure 3A, C, and D; Additional file 3: Figure S2). Protozoan GO term assignments, on the other hand, were usually reduced in “reinfected” tissue (Figure 3C, Additional file 3: Figure S2). Several categories in the metazoan GO term assignments showed more sequences derived from the “normal” treatment (e.g., multicellular organismal process, biosynthetic processes, gene expression, translation, generation of precursor metabolites and energy, ion transport, and mitochondrion organization - Figure 3A). GO assignments using the bacterial and the protozoan databases showed different trends (Figure 3; Additional file 3: Figure S2), but it is not clear how these relate to the presence or absence of *Symbiodinium* populations in *C. varians*. Attempting to discern how these interacting systems influence one another would provide an intriguing line of research, but goes beyond the capacity of the current study.

### Differential expression analysis

**Figure 4:**
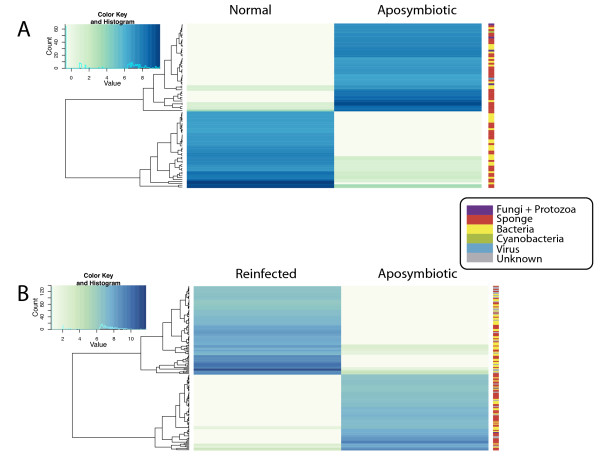
**Heatmaps and dendrograms comparing the differentially expressed genes between “normal” and “aposymbiotic” treatments (A) and “aposymbiotic” and “reinfected” treatments (B).** The affiliation of the different contigs showing differential expression to either Metazoa, Bacteria, Protozoa, Fungi, and Virus is shown in a color-coded bar next to each heatmap.

In the differential expression analysis using DESeq package [55] for the comparison between “normal” and “aposymbiotic” treatments, 87 genes showed significantly different expression values (Figure 4A; Additional file 2: Table S1). Forty-nine genes showed significantly higher expression in “aposymbiotic” tissue compared to “normal” tissue (30 of which were identified as coming from metazoan sources), while 38 genes were at significantly higher levels in “normal” tissue (19 of which were metazoan - Figure 4A; Additional file 2: Table S1). We found 160 genes that showed significantly different levels of expression when “aposymbiotic” and “reinfected” tissues were compared (Figure 4B; Additional file 2: Table S1). Eighty-six of the genes were at significantly higher levels in “reinfected” tissues (23 metazoan) while 74 were significantly higher in “aposymbiotic” tissue (42 metazoan - Figure 4B; Additional file 2: Table S1). While many genes were expressed at different levels in “reinfected” vs. “normal” tissue, DESeq analysis revealed no statistically significant differences in expression values.

**Figure 5:**
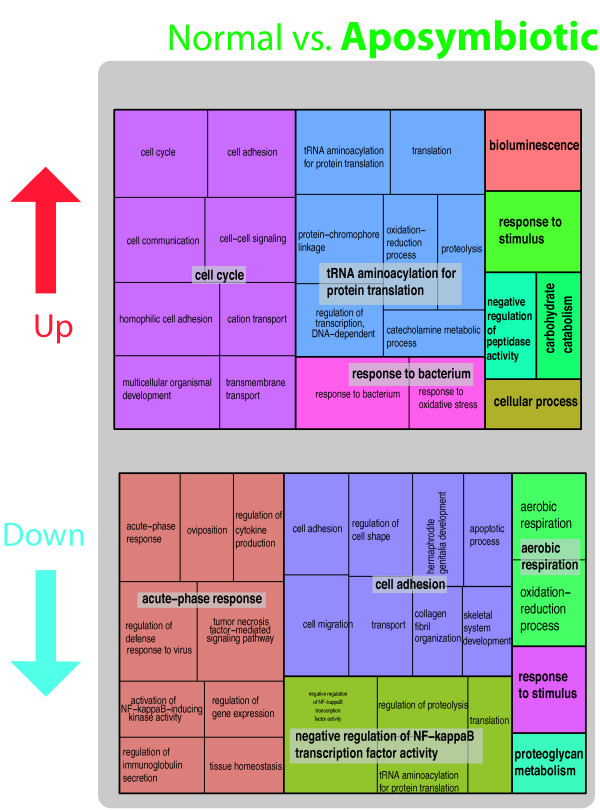
**Gene Ontology treemaps for the differentially expressed genes (both increased and decreased in expression) in the “aposymbiotic” treatment in the comparison “normal” vs. “aposymbiotic.”** GO terms for genes expressed in “aposymbiotic” tissue are shown. The box size correlates to the –log10 p-value of the GO-term enrichment. Boxes with the same color can be grouped together and correspond to the same upper-hierarchy GO-term which is found in the middle of each box.

Gene Ontology treemaps that display hierarchical data using nested rectangles (see [56]) reveal that some of the genes expressed at higher levels in the “aposymbiotic” treatment (compared to “normal”) were particularly interesting in the context of symbiosis (Additional file 4: Table S4; Figure 5 top). Within the broad “cell cycle” category, processes such as cell communication and signaling as well as trans-membrane transport may highlight a response by the host to the presence or absence of a putative symbiont. Another notable GO category had to do with “protein translation,” broadly defined, and included *DBH-like monooxygenase 1*, which is involved in the catecholamine metabolic process, and *sulfide:quinone oxidoreductase, mitochondrial-like* (SQR), which encodes an enzyme that oxidizes sulfide to thiosulfate. SQR is a potentially important enzyme because sulfide is produced endogenously in several tissues of marine invertebrates [57], and may be related to sulfide-oxidizing bacteria [58]. One explanation could be that the removal of *Symbiodinium* populations in the “aposymbiotic” treatment may have modified other components of the microbial consortia residing in sponges resulting in differential regulation of the host gene expression profile.

Interesting Gene Ontologies are also revealed when comparing genes expressed at higher levels in “normal” compared to “aposymbiotic” tissue (Figure 5 bottom) including members of the *TNF* family (e.g., *TNF receptor-associated factor 3-like*), which are important in immune responses (e.g., “acute-phase response” Figure 5 bottom). Other interesting genes included *deleted in malignant brain tumor* and *niemann pick c1* (Additional file 4: Table S4; Figure 5). These genes are discussed further below. It was intriguing that some of the genes that appear at higher frequency in “normal” tissue compared to “aposymbiotic” tissue (Additional file 4: Table S4 and Figure 5) are involved in “cell adhesion” (e.g., *collagen alpha-1(I)* chain, *basement membrane-specific heparan sulfate proteoglycan core protein-like*, and focal adhesion like *fibronectin*, which is an ECM component that acts as the *integrin* ligand [59]). This may relate to the movement and re-organization of *Symbiodinium*-bearing cells in mature symbiont populations.

**Figure 6:**
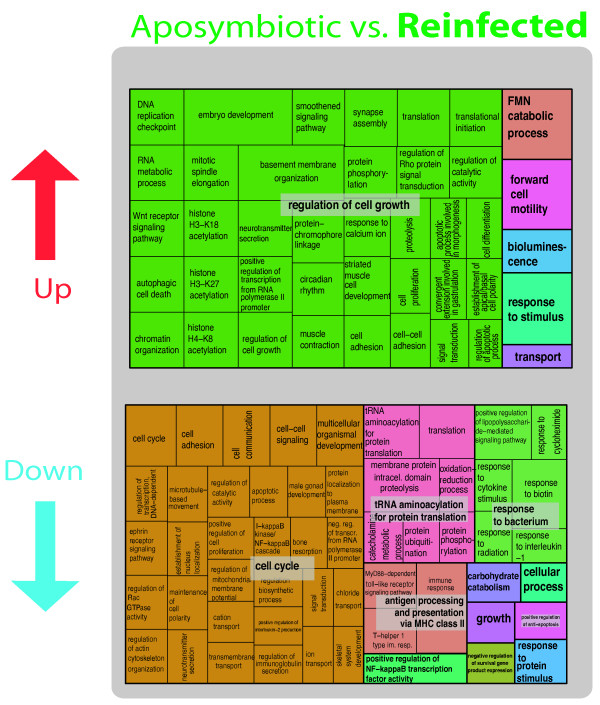
**Gene Ontology treemaps for the differentially expressed genes (both increased and decreased in expression) in the “reinfected” tissues for “aposymbiotic” vs. “reinfected” treatment comparisons.** The box size correlates to the –log10 p-value of the GO-term enrichment. Boxes with the same color can be grouped together and correspond to the same upper-hierarchy GO-term which is found in the middle of each box.

Most of the genes that had significantly higher expression levels in “reinfected” tissue compared to “aposymbiotic” tissue were involved in the “regulation of cell growth” (Figure 6 top). For example, *astacin* (Additional file 4: Table S4, Figure 6) is a metalloprotease involved in cell adhesion and pattern formation by processing extracellular proteins [60]. Two different transcripts with homology to *sarcoplasmic calcium binding protein* (Additional file 4: Table S4; Figure 6), which plays a role in calcium sequestration within endomembrane spaces, are interesting given our hypothesis that *Symbiodinium* spp. select hosts based on their ability to provide the dinoflagellate access to calcium stores (the magnesium inhibition hypothesis [13]). We also found that two genes containing the fibrinogen domain were increased in expression in “reinfected” tissue compared to “aposymbiotic” tissue (Additional file 4: Table S4, Figure 6); in invertebrates, the fibrinogen domain has been found to be associated with innate immunology and pathogen intolerance [61].

The treemaps provided unique insights into some of the patterns observed in our comparison of expression profiles in the different tissue types. The two panels that describe increased levels of expression in “aposymbiotic” tissue (Figure 5 top; Figure 6 bottom) showed very similar patterns in GO assignments. The top three categories for each of these comparisons were “cell cycle,” “tRNA aminoacylation for protein translation,” and “response to bacterium” (Figure 5 top; Figure 6 bottom). Some of the remaining categories were also identical (“carbohydrate catabolism” and “cellular process”). The situation was different for the other two comparisons that involved higher levels of gene expression in the presence of *Symbiodinium* (Figure 5 bottom; Figure 6 top). The differences in GO assignments here point to the possibility that different cellular processes are operating in a mature symbioses (“normal” tissue) compared to an association that is at an earlier stage of re-establishing *Symbiodinium* populations (“reinfecting” tissue). For example, “regulation of cell growth” was the predominant GO signature of genes that showed higher expression in “reinfecting” vs. “aposymbiotic” tissue. This broad category presents a suite of genes that would be worthy of future work to ascertain their importance in the development of a stable *Symbiodinium* symbiosis.

We found interesting patterns in global gene expression patterns among “normal”, “aposymbiotic”, and “reinfected” tissue treatments (Additional file 5: Figure S3). While the significant differences observed using the DESeq analysis described above are interesting, it is important to recognize that subtle differences in gene expression profiles that do not rise to the level of statistical significance estimated with a methodology like the one implemented in DESeq may still play important biological roles in regulating the interaction between partners in this symbiosis. Thus, closer inspection of specific GO categories provides important perspectives on the interplay that may occur between partners in this sponge: algal association. However, high throughput sequencing generates a large and complicated suite of genes and gene networks to consider, thus it is necessary to reduce the complexity of the dataset and identify testable hypotheses for future experiments. Therefore, we examined pathways that might relate to a recent hypothesis that posits that *Symbiodinium* spp. may mimic the phagosome by releasing materials at a rate and of a quality that would be expected from digesting prey thus securing their intracellular position [13]. This “arrested phagosome hypothesis (APH)” offers a subtly different perspective on the cellular machinations in operation when *Symbiodinium* take up residency in host cells. If *Symbiodinium* spp. use their photosynthetic capabilities to maintain residence within the intracellular habitat (but are “parasitic” in other aspects of their life history), then we may expect different types of genetic expression profiles than if the host is somehow controlling the association (e.g. [32, 62]). It is clear, however, that our non-replicated transcriptomes must be interpreted cautiously as trends we observed may not represent statistically significant differences.

**Figure 7:**
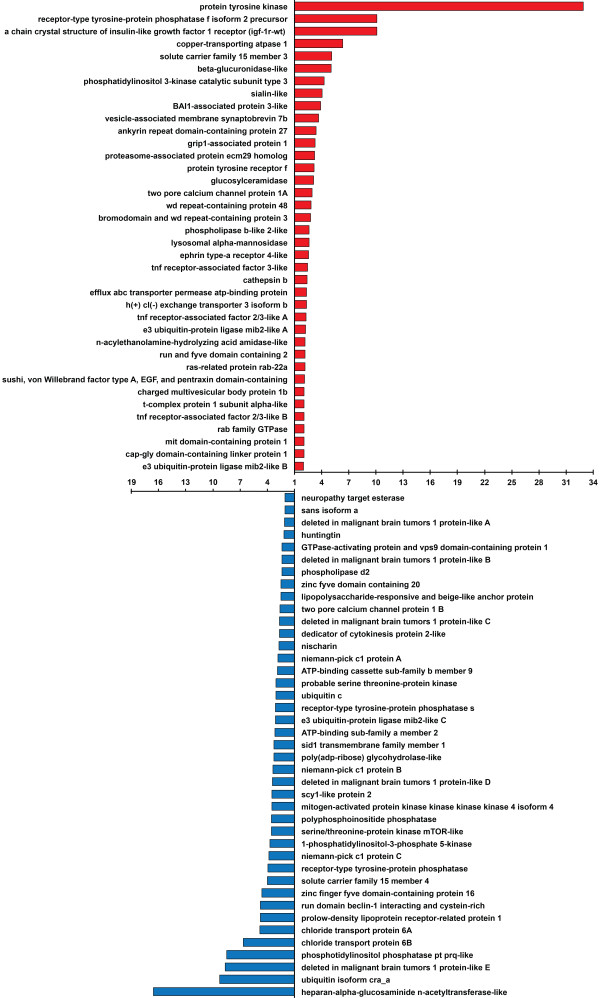
**Comparison of “reinfected” versus “aposymbiotic” expression patterns for GO categories related to endosome, lysosome, and phagosome function.** Red bars represent fold differences where genes appeared to be at least two-fold more common in the “reinfected” transcriptome compared to the same genes from the “aposymbiotic” transcriptome. Blue bars represent fold differences where genes appeared to be at least two-fold more common in the “aposymbiotic” transcriptome than the same genes found in the “reinfected” transcriptome.

The first group we considered included endosome, lysosome, and phagosome processes. For the endosome category we used GO:0005768 (and child nodes). For the lysosome category we used GO:00057864 (and child nodes). No single phagosome category was available so we used the following terms: phagocytic vesicle (GO:0045335), phagosome maturation (GO:0090382), phagocytosis, engulfment (GO:0006911), phagolysosome (GO:0032010), phagosome-lysosome fusion (GO:0090385), phagosome acidification (GO:0090383), phagocytic vesicle membrane (GO:0030670), and early phagosome (GO:0032009). This analysis produced 38 genes that appeared to be at least two-fold more highly expressed in “reinfected” compared to “aposymbiotic” tissue, whereas 41 genes appeared to be at least two-fold more highly expressed in “aposymbiotic” tissue compared to “reinfected” tissue. Differences between these groups included *RAB* and *TNF* family genes that were represented two-fold or higher in “reinfected” tissue compared to “aposymbiotic” tissue (Figure 7; Additional file 6: Table S2). Two groups of genes with several representatives each (*deleted in malignant brain tumor* and *niemann pick c1*) appeared to be represented at higher frequencies in “aposymbiotic” but not in “reinfected” tissue (Figure 7; Additional file 6: Table S2). If *Symbiodinium* spp. mimic endosomal structures, as predicted by the APH, the genes identified here are excellent candidates for future work aimed at manipulating expression profiles to pinpoint the cellular components used to gain access to the intracellular habitat. For example, RNAi techniques that permit reducing expression levels of particular genes have recently been developed for sponges (Rivera et al. [42]) and would be applicable to the *Cliona*:*Symbiodinium* association.

**Figure 8:**
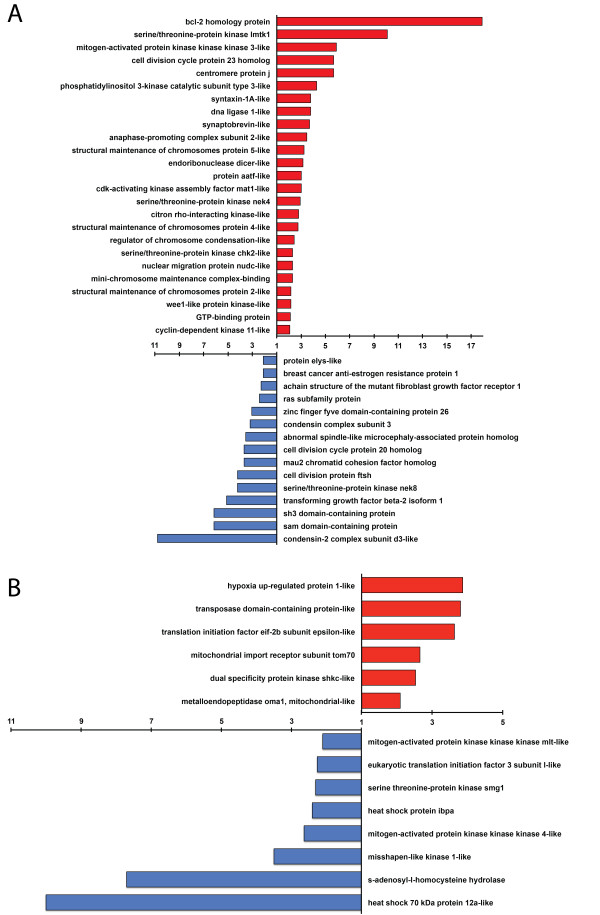
**Comparison of “reinfected” versus “aposymbiotic” expression patterns. A**. GO categories related to cell division are shown. Red bars represent fold differences where genes appeared to be at least two-fold more common in the “reinfected” transcriptome than the same genes in the “aposymbiotic” transcriptome. Blue bars represent fold differences where genes appeared to be at least two-fold more common in the “aposymbiotic” transcriptome than in the “reinfected” transcriptome. **B**. GO categories related to generalized stress are shown. Colors as in **A**.

*Symbiodinium* has been shown to energetically subsidize its *C. varians* host [10, 46]. Therefore, we examined a proxy for growth to see if any differences between “aposymbiotic” and “reinfected” tissue could be detected. We selected the GO category of cell division (GO:0051301 and child nodes). Twenty-five genes were at least two-fold more highly expressed in “reinfected” compared to “aposymbiotic” tissue whereas only 15 genes with those values were found in “aposymbiotic” compared to “reinfected” tissues (Figure 8A; Additional file 6: Table S2). It is interesting that *Bcl-2* and *condensin II* had the highest fold representation in reinfected and aposymbiotic tissue respectively. The Bcl-2 protein suppresses apoptosis by preventing the activation of caspases. The *condensin II* gene orchestrates chromosome condensation and thus helps regulate mitosis. Thus, these genes play vital roles in regulating the production of new cells, and the interplay that goes on upon reinfection indicates that the cellular dynamics are complicated. *Symbiodinium* spp. may benefit from the actions of these genes because a greater number of cells (and thus habitats) would be available for colonization.

In addition to the positive energetic benefits gained by hosts from their symbionts, *Symbiodinium* partners might also increase physiological stress on their hosts (e.g. [63]). It is also possible that by inoculating dark-acclimated aposymbiotic *C. varians* with a large dose of symbionts, and placing them under lighted conditions, we stressed the sponges involved in the reinfection experiments. Thus, we assessed generalized stress responses in “reinfected” compared to “aposymbiotic” tissue. We identified 6 genes involved in response to stress (GO:0006950) that were at least two-fold more common in “reinfected” compared to “aposymbiotic” tissue (Figure 8B; Additional file 6: Table S2). Using that same GO category, we identified 8 genes that were at least two-fold more common in “aposymbiotic” compared to “reinfected” tissue (Figure 8B; Additional file 6: Table S2).

### Suppressive subtractive hybridization

We also employed suppressive subtractive hybridization (SSH) technology as an alternative methodology to search for mRNA sequences represented in higher abundance in “reinfected” tissue compared with “aposymbiotic” *C. varians* tissue. We originated SSH before RNASeq technology became feasible for our study so the SSH experiments were not originally designed to complement the RNAseq experiments. However, we subsequently realized that the SSH data provided another avenue to verify some of the patterns we observed in the RNASeq experiments. We sequenced 173 clones recovered from our SSH analyses. Of these, 102 were not used for further analysis because they came from bacterial, protozoan or non-metazoan sources (n = 19), recovered no significant BLAST hits (n = 54; despite some showing metazoan-specific characteristics), were taxonomically-unaffiliated hypothetical/predicted proteins (n = 9), or lacked inserts/interpretable sequences (n = 17). Of the remaining 71 sequences, 15 sequences did not receive strong enough support during BLAST searches (blastx and tblastx) to assign a gene name with confidence. The remaining 56 clone inserts contained some redundancy (i.e., the same clone was pulled out of the library more than once), but the final 40 metazoan orthologs could be confidently identified based on gene orthology (Table 2). These genes should be expressed at higher levels with “reinfected” tissue, and thus afford an opportunity to independently verify for a subset of genes patterns we observed in the RNA-Seq experiments.

**Table 2:** **Results from suppressive subtractive hybridization experiments**

Gene name	Insert size (bp)	E-value	Function	GO terms	Clones
3-hydroxybutyrate dehydrogenase type 2	270	8.00E-17	degradation of ketone bodies	metabolic process, catalytic activity	1
Actin-related protein 2/3 complex subunit	698	2.00E-65	cell locomotion & phagocytosis	cytoskeleton, protein binding	1
AP-2 complex subunit beta	672	3.00E-49	clathrin-mediated endocytosis	membrane, transport, protein binding	1
ATPase, H+ transporting, lysosomal, V0 subunit	359	1.00E-28	acidification control	ion transport, transport	1
Ca2+-triggered coelenterazine-binding protein 2	542	1.00E-14	calcium ion binding	calcium ion binding	1
Calcium-binding protein p22; Calcineurin	366	5.00E-47	calcium-dependent phosphatase	signal transduction	1
CHK1 checkpoint-like protein	203	3.00E-29	kinase activity in mitosis	protein kinase activity, nucleotide binding	1
Creatine kinase U-type, mitochondrial	511	3.00E-48	energy production and transport	nucleotide binding, transferase activity	1
Cyclophilin A	297	2.00E-50	calcium inhibition	protein folding, hydrolase activity	1
Cyplasin S	270	4.00E-08	Cell death induction	oxidoreductase activity	1
Cytoskeletal actin	330, 370	2.00E-60, 2.00E-58	cell motility & maintenance	cellular protein metabolic process,	2
Deleted in malignant brain tumors 1 protein-like; Scavenger receptor cysteine-rich type protein	669, 906	9.00E-34, 3.00E-32	removal of foreign substances	membrane	2
Dihydrolipoyl dehydrogenase	352	3.00E-31	mitochondrial glycine cleavage	cytoplasm, glycolysis	1
Dynein heavy chain	630	3.00E-60	cellular transport & maintenance	biological process, transferase activity	1
Ephrin type-B receptor 1; Protein tyrosine kinase	404	2.00E-17	developmental regulation	nucleotide binding, transferase activity	1
Ferritin	358 - 721	9.00E-86 - 1.00E-18	iron storage	ion binding	3
Ficolin-2	357	3.00E-29	innate immune recognition	signal transduction	1
G-protein gamma subunit	514	2.00E-04	signal transduction	signal transduction	1
Gamma-interferon-inducible lysosomal thiol reductase like	386	5.00E-23	macrophage activation	catalytic activity, biological process	1
Glutamine synthetase	316	7.00E-23	nitrogen metabolism	cellular nitrogen compound metabolic process	1
Heat shock protein 70	546	5.00E-24	protein folding & stress protection	response to stress	1
Hypothetical proteins	389 - 564	8.00E-24 - 2.00E-04	calcium absorption & metabolism		3
Inorganic pyrophosphatase	421	7.00E-41	lipid metabolism & calcium absorption	cytoplasm, ion binding	1
MafB chain A	428	5.00E-26	hematopoiesis regulation	transcription, cell death	1
Neurogenic locus notch protein homolog	617	5.00E-05	proliferative signaling	signal transduction	1
Nuclear pore complex Nup50	687	2.00E-29	intracellular protein transport	carbohydrate metabolic process	1
Proteasome subunit alpha	573	2.00E-26	processing of MHC class I peptides	cellular nitrogen compound metabolic process	1
Proteasome subunit beta	464	1.00E-64	intracellular protein degradation	cellular protein metabolic process, gene expression	1
Ribonuclease K-like; Salivary secreted ribonuclease	556	3.00E-14	degredation & protection	transport	1
Ribosomal proteins	223 - 383	3.00E-37 - 2.00E-04	translation machinery	translation, cellular protein metabolic processes, gene expression	8
RNA polymerase-associated protein LEO1	500	3.00E-07	histone methylation	protein binding, transcription	1
Selenoprotein Jb; J1a crystallin	637	1.00E-21	regulation of metabolism	hydrolase activity	1
Serum response factor	686, 709	9.00E-07, 6.00E-06	developmental regulation	cytoskeleton, signal transduction	2
Sulfide quinone reductase	259	5.00E-14	oxidation catalysis	oxidoreductase activity	1
Thymosin beta	295	2.00E-07	actin-sequestering protein	cytoskeleton	1
Tubulin alpha chain	283	2.00E-12	microtubule assembly	cytoskeleton	1
Tyrosine 3-monooxygenase/tryptophan 5-monooxygenase activation protein	482	2.00E-53	phosphoserine-binding for signal transduction	cytoplasm, protein targeting	1
Vacuolar sorting protein; sortilin-related receptor	789	1.00E-29	neuropeptide receptor activity & protein binding	membrane	1
von Willebrand factor A domain-containing protein-5a	507	2.00E-07	intracellular ligand interactions	transport	1
WAS protein family homolog 1	535	4.00E-11	nucleation promoting factor on endosomal surface	transport	1

BLAST searches for each isolate (insert sizes shown) against NCBI database were used to determine gene identity. Gene function and GO categories were inferred from gene identity.

**Figure 9:**
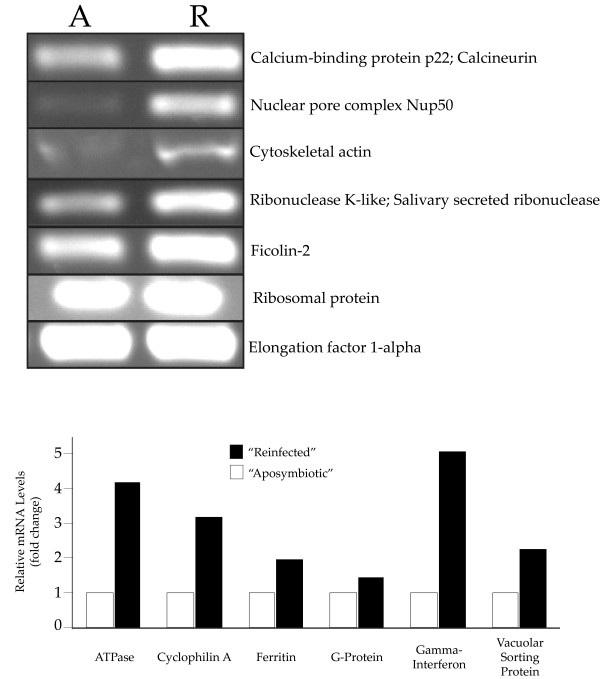
**RT-PCR validation of relative (fold) expression differences for representative genes isolated by suppressive subtractive hybridization when comparing mRNA from “aposymbiotic” tissue to mRNA from “reinfected” tissue (top: gel electrophoresis of RT-PCR products, bottom: qRT-PCR).** qRT-PCR expression values were normalized to the housekeeping gene EF1a.

We tested these genes as a possible validation of expression patterns observed in the transcriptomic datasets. We compared the raw reads obtained from the RNA-Seq data to each clone identified via SSH (Table 2), which should come from genes up-regulated in “reinfected” compared to “aposymbiotic” tissue. Over 80% of the contigs in the transcriptome that aligned with our SSH clones showed a trend of increased expression in “reinfected” tissue compared to “aposymbiotic” tissue as expected (Additional file 7: Table S3). None of the SSH genes were expressed at lower levels in “reinfected” tissue compared to “aposymbiotic” tissue. However, 9 SSH clones (16.1% of all clones) that corresponded to 7 of the SSH genes revealed contig expression patterns that were both higher and lower in “reinfected” compared to “aposymbiotic” tissue. Thus, we are unable to confirm that these genes show increased expression upon uptake of *Symbiodinium* (Additional file 7: Table S3). It is important to note, however, that these results are expected given that SSH methods using PCR-Select cDNA Subtraction (Clontech) have a false positive rate that is caused by the presence of remnant cDNAs common to both tester (“reinfected) and driver (“aposymbiotic) samples. We also note that possible false positives in this type of SSH can depend can depend on a variety of factors including RNA quality, mRNA abundance, and performance of the subtraction. This was, to our knowledge, the first time this technique has been applied to poriferan systems, and we had no other studies to compare our efficiencies. We also validated the differential expression patterns we observed for a subset of the animal-specific clones by RT-PCR followed by gel electrophoresis and/or relative qRT-PCR (Figure 9). Of the genes with higher expression values in *C. varians* upon infection with *Symbiodinium*, we found that several genes that play documented roles in host phagosomes (e.g., Vacuolar sorting protein, Nup50, calcium-binding protein) and host immune responses (ficolin, gamma-interferon-inducible lysosomal protein) were represented (Table 2). We highlight, however, that any candidate genes identified in this study (by SSH or transcriptomic analysis) should be subjected to rigorous evaluation on a gene-by-gene basis in controlled and natural re-infection experiments with multiple biological replicates, as well as in functional experiments, before their role(s) in *Symbiodinium*-symbioses can be confirmed and delineated.

### Field experiments

**Figure 10:**
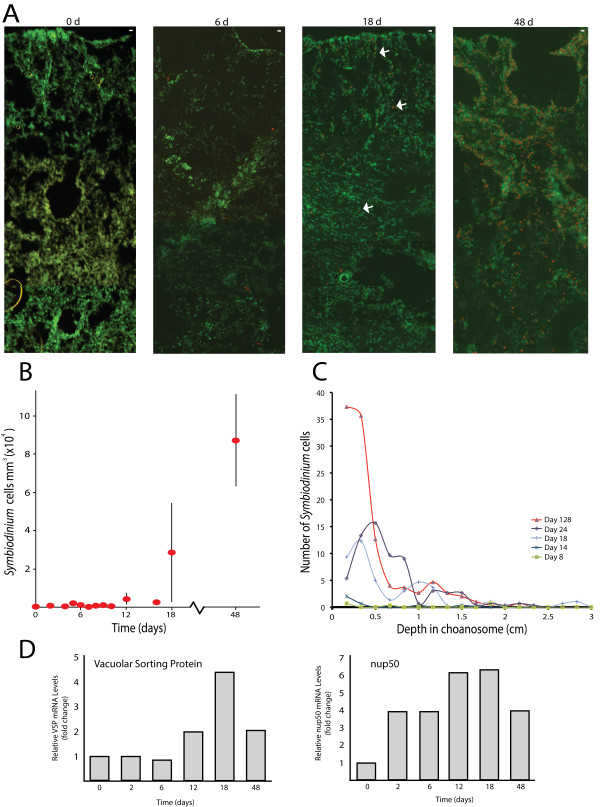
**Temporal dynamics of** ***Symbiodinium*** **reinfection of aposymbiotic** ***Cliona varians*** **tissue in under field conditions. A**. Cryosections through sponge tissue starting at the pinacodermal (i.e., external) surface of the sponge down through the choanosome. Red/orange dots represent *Symbiodinium* cells. Scale bar (upper right corner of each figure) = 10 μm. **B**. Estimates of *Symbiodinium* density for the time points collected during the reinfection experiment. **C**. Density of *Symbiodinium* as a function of depth within the sponge tissue. **D**. Expression profiles for two genes (*NUP50* and *vacuolar sorting protein*) as a function of time (and thus symbiont density). Y-axis represents the fold change in gene expression relative to time 0 with all points normalized to the housekeeping gene *EF1α*.

We were interested in assessing the utility of the *C. varians* system as a means to examine genetic interactions between host and *Symbiodinium* under field conditions. Few studies have correlated gene expression profiles with symbiont population dynamics under ecologically realistic conditions, and we were interested in determining whether we could do this using genes identified above. Thus, our natural reinfection experiment may represent a methodological advance in *Symbiodinium* research. *Cliona varians* provides a useful model to study temporal aspects of reinfection dynamics in *Symbiodinium* associations because we demonstrate algal densities and locations can be monitored precisely. We detected very few *Symbiodinium* cells in *C. varians* tissue during the first 7 days in the field (Figure 10). By the 8^th^ day, we observed a small number of *Symbiodinium*-like cells, and after the 12^th^ day the symbionts repopulated aposymbiotic sponges at a nearly exponential rate (Figure 10B). The presence of the algae within the tissue was skewed towards the surface layer after 1 month in the field (Figure 10C). After 16 d, the symbionts rapidly increased their populations and recovered nearly normal concentrations of *Symbiodinium* by 128 d. We typed the *Symbiodinium* populations using 23S rDNA sequences (see [13]) and exclusively found G Clade algae (data not shown). As a test of this experimental system, we correlated gene expression profiles for *vacuolar sorting protein* and *NUP50* (given their possible roles in an arrested phagosome) with *Symbiodinium* population dynamics within the host. We identified interesting temporal patterns in gene expression using qRT-PCR. Expression patterns differed for each gene at days 0, 2, 6, 12, 18, and 48 (Figure 10D). It was particularly intriguing that *vacuolar sorting protein* had elevated expression near the onset of rapid *Symbiodinium* population growth (i.e., around day 18). This represents the earliest stages of discerning nuanced aspects of host:symbiont integration at the genetic level. Additional experiments on other key genes are needed, but our results indicate that the tools are available to precisely describe the nature of the symbiosis at the finest level of genetic resolution.

## Conclusions

Our results add to the growing perspectives on molecular genetic integration between hosts and symbionts in *Symbiodinium*-based associations. This is, however, the first that provides insights into the genetic pathways that appear to be important in poriferan: *Symbiodinium* partnerships. Our results indicate that hosts, regardless of taxonomic origin, engage similar cellular and genetic processes in response to intracellular zooxanthella-residency [25–31, 33, 64]. High-throughput sequencing offers opportunities to generate massive datasets, and we found that comparing the transcriptomic data with results generated through suppressive subtractive hybridization provided an interesting mechanism to validate a portion of our non-replicated RNASeq data. The RNA-Seq experiments and cross-validation with an independent methodology (e.g., SSH) provide confidence that we have identified some appropriate candidate genes for future work focused on detailing precise genetic regulation of symbiont and host interactions. However, any differences observed in the present study should be treated cautiously since they come from transcriptomes that were not replicated within treatments. One of our goals was to demonstrate the importance of integrating ecologically-relevant scenarios with insights gained through acquisition of lab-based gene expression data. Sponges may be exceptionally useful systems of study in this context. Specifically, the temporal variability seen in expression dynamics under natural conditions in the field highlight how nuanced the interaction between the host and symbiont is likely to be, and how much work remains to uncover detailed perspectives on the associations.

Through this and related work, it appears possible to identify some common pathways that *Symbiodinium* may co-opt to gain entry and to procure residency in a variety of potential hosts. Nonetheless, clear explanatory hypotheses are needed so that we can better understand, and prepare for, changes in the symbiosis that are likely with the rapid shifts in temperature and sea-water chemistry that will accompany global climate change [13, 65, 66]. We also require more detailed knowledge of the interaction between symbiotic partners. We argue that sponge: *Symbiodinium* associations add important perspectives on the *Symbiodinium* niche, which will foster greater understanding in other host environments.

## Methods

### Creation of aposymbiotic and reinfected sponges

*Cliona varians* forma *varians* were collected from shallow (≈1 m) flats just south of the Mote Tropical Research Laboratory in Summerland Key, FL (24.658, −81.452). All collections performed in the Florida Keys for this study were obtained with all appropriate and relevant permits and licenses. In accordance with policies established by the Florida Keys National Marine Sanctuary, we collected sponges under permit FKNMS-20070094-A1 and under a Florida recreational resident saltwater fishing license issued from Florida Fish and Wildlife Conservation Commission. Sponges were transported to shallow raceways where the *Symbiodinium*-dense pinacodermal region was removed with a sharp razorblade (Additional file 1: Figure S1). The *Symbiodinium* devoid choanosome explants were placed in a lightproof container (≈60 L total volume) to heal for several months where they received fresh seawater from an underground aquifer, which is unlikely to contain free-living *Symbiodinium*, at a rate of approximately 2 L min^−1^. Small explants (≈6-8 cm^3^) of the “aposymbiotic” sponges were then exposed to *Symbiodinium* that had been freshly isolated from *C. varians* forma *varians* (Figure 1). After 5 days, signs of reinfection were visible to the naked eye (Figure 1). At this point, tissue was harvested, placed in 1.5 ml tubes, flash-frozen in liquid nitrogen, and immediately stored at −80°C until mRNA was extracted.

### Transcriptome sequencing

For transcriptomic analysis, mRNA was isolated directly from the tissue samples (three biological replicates of each tissue type were pooled within the same tube) using the Micro-FastTrack 2.0 mRNA isolation kit (Invitrogen), and mRNA samples from the replicates were pooled. Quantity and quality (purity and integrity) of mRNA were assessed by three different methods. We measured the absorbance at different wavelengths using a NanoDrop ND-1000 UV spectrophotometer (Thermo Fisher Scientific, Wilmington, Massachusetts, USA). Quantity of mRNA was also assessed with the fluorometric quantitation performed by the QubiT® Fluorometer (Invitrogen, California, USA). Also, capillary electrophoresis in an RNA Pico 6000 chip was performed using an Agilent Bioanalyzer 2100 System with the “mRNA pico Series II” assay (Agilent Technologies, California, USA). Integrity of mRNA was estimated by the electropherogram profile and lack of rRNA contamination (based on rRNA peaks for 18S and 28S rRNA given by the Bioanalyzer software). We used the TruSeq RNA Sample Prep Kit (Illumina, Inc.) to prepare the three different library samples of *C. varians* using 135.8 ng of mRNA for the normal tissue, 665 ng for the aposymbiotic tissue, and 743.5 ng for the reinfected tissue following the manufacturer’s instructions with minor modifications. Fragmentation was performed on mRNA for 1.5 min, and fragments of 350 bp were targeted through size selection on excised gel bands of 2% agarose. The three samples were multiplexed using Index 4 for the normal tissue, 6 for the aposymbiotic tissue, and 12 for the reinfected tissue from the TruSeq RNA Sample Prep Kit.

The concentration of the cDNA libraries was measured with the QubiT® dsDNA High Sensitivity (HS) Assay Kit using the QubiT® Fluoremeter (Invitrogen, Carlsbad, California, USA). The quality of the library and size selection were checked using the “HS DNA assay” in a DNA chip for Agilent Bioanalyzer 2100 (Agilent Technologies, California, USA). cDNA libraries were considered successful when the final concentration was higher than 1 ng μl^−1^ and the bioanalyzer profile was optimal [50]. We obtained 1.065 μg of cDNA for the normal tissue, 0.45 ng for the aposymbiotic tissue, and 0.09 ng for the reinfected tissue. The libraries were brought to 10 nM prior to sequencing. Next-generation sequencing was performed using the platform Illumina HiSeq (Illumina, Inc., San Diego, California, USA) at the FAS Center for Systems Biology at Harvard University. The normal and aposymbiotic treatments were run together with another invertebrate library in one lane, and the reinfected treatment was run in another lane with two more invertebrate libraries. Paired-end reads were run to 101 bp.

### Transcriptome assembly and annotation

Trimming analyses for the raw reads of each independent transcriptome dataset were done with CLC Genomics Workbench 5.1 (CLC bio, Aarhus, Denmark). Initial trimming was performed using 0.5 as the limit of the quality score (based on Phred quality scores), and resulting quality of the trimmed reads was visualized with FastQC (http://www.bioinformatics.bbsrc.ac.uk/projects/fastqc/). After this, only those terminal bases with a Phred quality score under 30 were trimmed (where a Phred score of 30 corresponds to a probability of 1 in 1,000 of incorrect base calling), which produced sequences of unequal size. High-quality reads were re-screened to check for presence of adapter or primer sequences using FastQC, and if present, they were removed using with CLC Genomics Workbench 5.1.

Four *de novo* assemblies were performed using CLC Genomics Workbench 5.1: three separate assemblies containing the raw reads of each treatment, and another one pooling all raw reads (called “reference”). Global alignments for the *de novo* assemblies were always done using the following default parameters: mismatch cost = 2; insertion cost = 3; deletion cost = 3; length fraction = 0.5; similarity = 0.8; and randomly assigning the non-specific matches. Best *k*-mer length was estimated by the software. The best assembly for each treatment was selected using an adaptation of the optimality criteria for *de novo* assembly with 454 data [50].

From the “reference” transcriptomic dataset, contigs shorter than 300 bp were removed (assuming that shorter contigs would retrieve very few results during blast searches). For the remaining contigs we performed BLAST searches against a database of selected proteins from the *nr* NCBI database (containing Metazoa, Bacteria, Fungi, Virus, and Protozoa, including *Symbiodinium* spp.). Since sponges host a wide variety of symbiotic organisms within their tissues, mainly bacteria and protozoans, that cannot be completely removed prior to cDNA construction, we performed separate BLAST searches against three different individual databases containing proteins of Metazoa, Protozoa, and Bacteria, to estimate the amount of contigs belonging to either symbionts or the sponge. Such searches were performed for the “normal”, “aposymbiotic”, and “reinfected” transcriptome datasets and the contigs showing hits against two or all the databases were counted. All BLAST searches were conducted with BLAST v2.2.23+ [67] using an e-value cut-off of 1e-5. With the resulting file, we then used Blast2GO v2.5.0 (Conesa et al. [68]) to retrieve the Gene Ontology (GO) terms and their parents associated with the top BLAST hit for each sequence. For the metazoan hits, we performed a Fisher’s exact test with multiple test correction by Benjamini–Hochberg false discovery rate (FDR) to analyze the differential GO term enrichment (P > 0.05) in each treatment.

### RNAseq analysis and differential expression

Only contigs of 1000 bp or longer from the “reference” transcriptome were used as a mapping reference for the evaluation of expression values because they were, in the majority of cases, assigned BLAST and GO term annotations. Quality trimmed reads from each of the three treatments were mapped against the “reference” dataset with CLC Genomics Workbench 5.1 as a short read aligner. The total number of unambiguously mapped reads (i.e., “unique genes”) of each treatment compared to the “reference” transcriptome was exported as a table to use as count data in further analyses. Differential expression values were computed with the DESeq package [55] in Bioconductor in R. We performed three different comparisons to find genes up-regulated in each treatment: “normal” versus “aposymbiotic”, “aposymbiotic” versus “reinfected”, and “normal” versus “reinfected”. We first estimated the effective library size, and then estimated the data’s dispersion and mean to identify differentially expressed genes. Due to the lack of non-pooled biological replicates for transcriptome sequences, we instructed the program to ignore the condition (i.e., “treatment”) labels and estimated variance by treating all samples as if they were replicates of the same condition. This approach follows that outlined in Anders [55]. Comparisons were accepted to be significant at an FDR adjusted value of 0.01. Only significant values were plotted as a heatmap using the R heatmap.2 function from the R ‘gplots’ library. We used the default hclust hierarchical clustering algorithm to cluster the rows. Finally, the affiliation of differentially expressed contigs to either Metazoa, Bacteria, Protozoa, Fungi, and Virus was obtained from BLAST results of the “reference” transcriptome.

We performed two enrichment analyses for the differentially expressed genes for which we obtained significant p-values and were also able to find associated GO terms (obtained in the annotation with Blast2GO of *de novo* assembled “reference” transcriptome). The enrichment analyses were performed for this set of differentially expressed genes using all three possible comparisons (“normal”, “aposymbiotic”, “reinfected”) by testing the up-regulated genes in one treatment against up-regulated genes in the other treatment. Enriched GO-terms were then slimmed in REVIGO and treemaps were produced (following [56]). We also conducted overall comparisons of the expression profiles of the three *C. varians* treatments. In addition, for overall comparisons of the expression profiles of the three treatments of *C. varians*, heat maps were obtained with CLC Genomics Workbench 5.1 by mapping the raw reads of each treatment dataset against the total “reference” contig list (292,182 contigs). Contigs of the “reference” dataset whose size exceeded 1000 bp (N = 15,636 sequences) were represented in detail to ensure full length. Expression was measured in RPKM (Reads Per Kilobase of exon model per Million mapped reads). Since no reference genome is available for *C. varians*, exons were not annotated for the analysis, and in turn, the assembled contigs were assigned a complete exon. We generated another heat map that included genes that had differences between RPKM among treatments of 2 (N = 13,773 sequences). All these analyses were performed without replication, and thus the results should be taken as a preliminary assessment of the gene expression profile of the tissues under the treatments.

Assuming that the transcriptome dataset “reference” contained most of the sponge genes present in the genome, we also estimated the ortholog hit ratio (OHR) as defined by O'Neil et al. [69]. The OHR describes the percentage of an ortholog “found” in a contig by dividing the number of non-gap characters in the query hit by the length of the subject using a script provided by Ewen-Campen et al. [54]. The workflow used to analyze all our transcriptomic data was provided by Riesgo et al. [50].

We compared the expression values to identify contigs that had either the highest or lowest occurrence in the “reinfected” tissue compared to the “aposymbiotic” tissue types. These values were reported as fold increase. It is important to note that we cannot assign significance to these differences – they are meant to demonstrate how candidate genes might be first identified. To narrow the large universe of genes that could possibly be examined, we focused our attention on GO terms that may be associated with pathways related to recently proposed hypotheses [13]. We truncated our analysis to genes that showed a 2-fold or higher difference between reinfected and aposymbiotic tissue.

### Suppressive subtractive hybridization

Suppressive subtractive hybridization (SSH) was performed using the Clontech PCR-Select cDNA Subtraction Kit®, following the manufacturer’s protocol. Poly A + mRNA was isolated from three biological replicates of *C. varians* aposymbiotic and reinfected tissue using the Micro-FastTrack 2.0 mRNA isolation kit (Invitrogen) and pooled before cDNA synthesis of RNA, which was performed using the Super Smart cDNA Synthesis Kit (Clontech). cDNA from reinfected tissue was used as the tester and cDNA from aposymbiotic tissue was used as the driver for the forward subtraction reactions. PCR products generated from the subtracted library, representing mRNAs putatively over-expressed in reinfected tissue, were sub-cloned into the TOPO TA cloning vector using OneShot TOP10 competent cells (Invitrogen) and plasmids were prepared using the QIAprep Spin Miniprep kit (Qiagen). Sequencing of 173 individual clones from the subtracted library was performed on an ABI 3130 × L Genetic Analyzer at Virginia Commonwealth University’s sequencing facility. Sequences were searched using the blastx and tblastx algorithms in the Genbank database. To validate that a subset of the identified genes were differentially expressed, RNA was isolated from aposymbiotic and reinfected *C. varians* using the RNeasy® Mini Kit (Qiagen), limiting genomic DNA contamination through an additional on-column DNase I treatment. cDNA was synthesized from equal amounts of sponge mRNA (125–200 ng/μl) using Superscript III reverse transcriptase (Invitrogen) and oligodT primer. In some cases, RT-PCR was conducted followed by gel electrophoresis to allow visual inspection of differential gene expression. In other cases, SYBR Green (Invitrogen) chemistry and Chromo4 (BioRad) were used to obtain relative levels of expression by qRT-PCR. Expression levels were normalized to the housekeeping gene *Ef1a* that qRT-PCR showed to be consistently expressed at high levels in both sets of tissues. For all qRT-PCR experiments, duplicates were performed from master mixes, and in most cases each experiment was repeated twice. Threshold values for Ct calculation were manually selected for all samples by placing the threshold line at the intersection where the signal intensities of the fluorescence traces surpassed background levels and began to increase (i.e., the linear portion of the curve). Both data and standard graphs were considered when establishing the position of the threshold line to optimize efficiency. Reaction efficiencies were recorded as efficiency per well in the linear range of the Ct and two points above. Standard curves, using plasmid dilutions of known quantities as templates, were generated for each gene in each qPCR experiment. Efficiency-corrected Ct values were compared to these curves (based on log of standard DNA concentration vs. Ct value for each sample) to calculate relative concentrations of samples using Opticon Monitor software (BioRad). The relative concentration values of duplicates were averaged and experimental averages were normalized to *Ef1a* values.

We used the SSH library as a partial validation of the gene expression values observed in the transcriptomic analysis. We first used the BLAST algorithm to search the transcriptome for transcripts matching our SSH clones. We used BLAST to verify GO terms and thus gene identity for contigs identified as having significant overlap with the SSH clone. In two cases, none of the contigs recovered the gene identified in SSH (i.e., cyplasin, a ribosomal protein). For the other 54 genes, we could verify contigs that aligned with our SSH gene. In some cases more than one contig aligned with the SSH clone so we examined the expression levels for each contig aligning with our SSH clone.

### Experimental analysis of gene expression profiles

A natural reinfection experiment was conducted in the flats south of Mote Tropical Research Laboratory (24.6605, −81.4551). Forty-two aposymbiotic *C. varians* explants were transplanted from their lightproof container into shallow water (>1 m). Explants were secured to a sheet of fiberglass window with monofilament. The window screen with sponges was situated on top of the substratum in an area populated with several potential *Symbiodinium* donors (e.g., *Porites divericata*, *Siderastrea radians*, *Cassiopea xamanchana*, and *Cliona varians*).

Preliminary experiments indicated that populations of intracellular *Symbiodinium* began to appear in aposymbiotic *C. varians* transplants after approximately six days in the field. Thus, from May to July, 2012, we sampled 3 explants from the field at 0, 2, 4, 5, 6, 7, 8, 9, 10, 12, 14, 16, 18, and 48 days post transplantation. The explants were transported to the lab within 30 min of collection where they were immediately processed for subsequent work. One section of each explant was immediately placed in RNA*later* RNA Stabilization Reagent from QIAGEN (for gene expression analyses), and stored overnight at 4°C. The next morning, RNA*later* was drained from the tube, and the tissue was frozen and stored at −80°C. A second section was snap frozen for DNA isolation. Another two sub-sections were taken from each explant and fixed in either a 4% paraformaldehyde: 2.5% gluteraldehyde solution (for electron and light microscopy work) or a 3.7% formaldehyde solution (for zooxanthella cell counts). Tissues were stored at 4°C, and after 24 h, the gluteraldehyde-containing samples were transferred to filter sterilized seawater and stored at 4°C until embedding, sectioning and visualization. Three randomly chosen *C. varians* individuals were sampled from the flats to serve as controls and were processed in the same manner described above. Differential expression of two genes (nup50 and vacuolar sorting protein) as a function of time post-transplantation was assessed by qRT-PCR as described above, however, expression values are plotted relative to time 0 after normalization to the housekeeping gene Ef1a. We selected nup50 and vacuolar sorting protein because they showed strong levels of up-regulation in reinfecting tissue, and thus represented robust candidates to demonstrate that this empirical approach would be a useful tool to test gene expression hypotheses generated by the transcriptome and SSH databases.

Paraformaldehyde: gluteraldehyde-fixed samples were embedded in OCT™ medium, frozen in liquid nitrogen, and sectioned with a Leica CM1850 cryostat at a thickness of 10 μm. Sections were stained with SYBR® green (1 μg μl^−1^) in 80% glycerol, and imaged using a Hamamatsu ORCA-ER camera attached to an Olympus BX61 microscope with a DG4 fluorescent lamp. *Symbiodinium* were visualized with a TX-RED filter (936 ms exposure) while SYBR green-stained nuclei could be distinguished using the FITC filter (1302 ms exposure). *Symbiodinium*-depth within sponge tissue was determined by stitching together successive images starting at the pinacoderm and moving deeper into the choanosome with Adobe Photoshop. Algal cells were counted in triplicate along microtransects (5 μm by 10 μm) that ran 3 cm into the choanosome. Total *Symbiodinium* cell counts were performed with formaldehyde-fixed samples. A block of known dimensions was cut from the pinacoderm into the choanosome. The tissue was ground with a mortar and pestle and the resultant slurry was suspended in 5 ml of filter-sterilized seawater. *Symbiodinium* cell concentrations were measured with a 0.1 mm deep Bright-line® hemacytometer. Five independent samples were taken from the suspension to calculate average zooxanthellae densities (cells mm^−3^ sponge tissue). DNA was isolated from frozen samples using a modified CTAB protocol and used in PCR reactions to amplify 23S rDNA [38]. PCR products were gel purified (Qiagen) before being sent to VCU’s DNA sequencing facility. Using BLAST, sequences were compared to NCBI’s nucleotide collection database to determine identity.

### Availability of supporting data

Transcriptomic sequences were deposited in the NCBI Sequence Read Archive. The experiment accession numbers for the raw reads deposit is as follows: “normal”: SRX333053, “aposymbiotic”: SRX333054, and “reinfected”: SRX333055. The Bioproject accession number for the whole project is: PRJNA214560, and the Biosample accession number is: SAMN02304131.

## Electronic supplementary material

Additional file 1: Figure S1: Extraction of *Cliona varians* choanosome involved cutting away the *Symbiodinium*-rich pinacoderm with a razor blade. The resulting choanosomal explant was nearly *Symbiodinium*-free (based on visual inspection), and was placed in a light-tight container with continuously flowing water for several months before use in the experiment. Pinacodermal tissue was returned to the environment to recover. (PNG 2 MB)

Additional file 2: Table S1: Contigs from all sources (e.g., metazoan, bacterial, and protozoan) that showed significantly different expression values in the “normal” vs. “aposymbiotic” and “aposymbiotic” vs. “reinfected” treatment comparisons (from Figure 4). Adjusted p values and protein names are shown. (PDF 83 KB)

Additional file 3: Figure S2: A. GO term assignment for the biological process category in each transcriptomic dataset (“normal”, “aposymbiotic”, and “reinfected” treatments) when using the databases Metazoa, Bacteria, and Protozoa (subselections of the nr database from NCBI). B. GO term assignment for the molecular function category in each transcriptomic dataset (“normal”, “aposymbiotic”, and “reinfected” treatments) when using the databases Metazoa, Bacteria, and Protozoa (subselections of the nr database from NCBI). C. GO term assignment for the cellular component category in each transcriptomic dataset (“normal”, “aposymbiotic”, and “reinfected” treatments) when using the databases Metazoa, Bacteria, and Protozoa (subselections of the nr database from NCBI). (ZIP 2 MB)

Additional file 4: Table S4: Metazoan contigs that showed significantly different expression values in the “normal” vs. “aposymbiotic” and “aposymbiotic” vs. “reinfected” treatment comparisons (from Figure 4). Adjusted p values and protein names are shown. (PDF 48 KB)

Additional file 5: Figure S3: Heat maps showing the expression levels of the total dataset, a subselection of contigs over 1,000 bp, and a subselection of contigs showing a difference of 2. (PDF 354 KB)

Additional file 6: Table S2: List of the contig assignments for each of the genes represented in Figures 7 and 8. (PDF 51 KB)

Additional file 7: Table S3: Comparison of expression levels found in the RNA-Seq experiment for each of the clones pulled out of the Suppressive Subtractive Hybridization library. SSH clone names are provided as are contigs that align to that sequence. The expression levels recorded from the transcriptome are indicated as are the fold differences. Numbers greater than one indicate that the “reinfected” expression is higher than the “aposymbiotic” expression. Numbers less than one indicate that the “aposymbiotic” expression is higher than the “reinfected” expression. (PDF 51 KB)
