# Supplementary figures and images for: Transcriptomic analysis of differential host gene expression upon uptake of symbionts: a case study with Symbiodinium and the major bioeroding sponge Cliona varians
Source: BMC Genomics. 2014 May 16;15(1):376. doi: 10.1186/1471-2164-15-376 (PMC4144087; doi:10.1186/1471-2164-15-376)

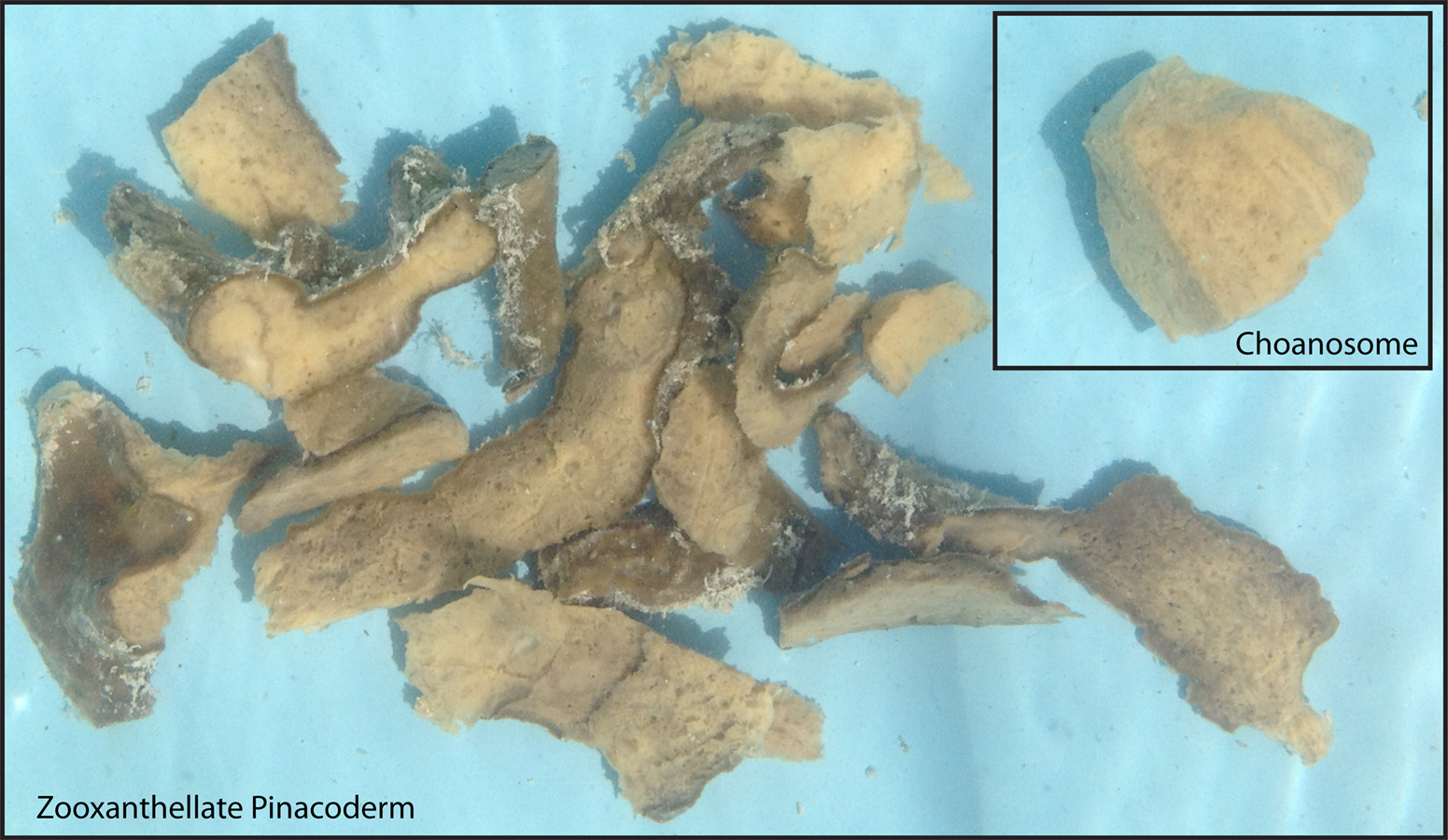

Supplement: Supplementary file 1 — Additional file 1: Figure S1: Extraction of Cliona varians choanosome involved cutting away the Symbiodinium-rich pinacoderm with a razor blade. The resulting choanosomal explant was nearly Symbiodinium-free (based on visual inspection), and was placed in a light-tight container with continuously flowing water for several months before use in the experiment. Pinacodermal tissue was returned to the environment to recover. (PNG 2 MB) [file 12864_2013_6178_MOESM1_ESM.png]

Biological Process

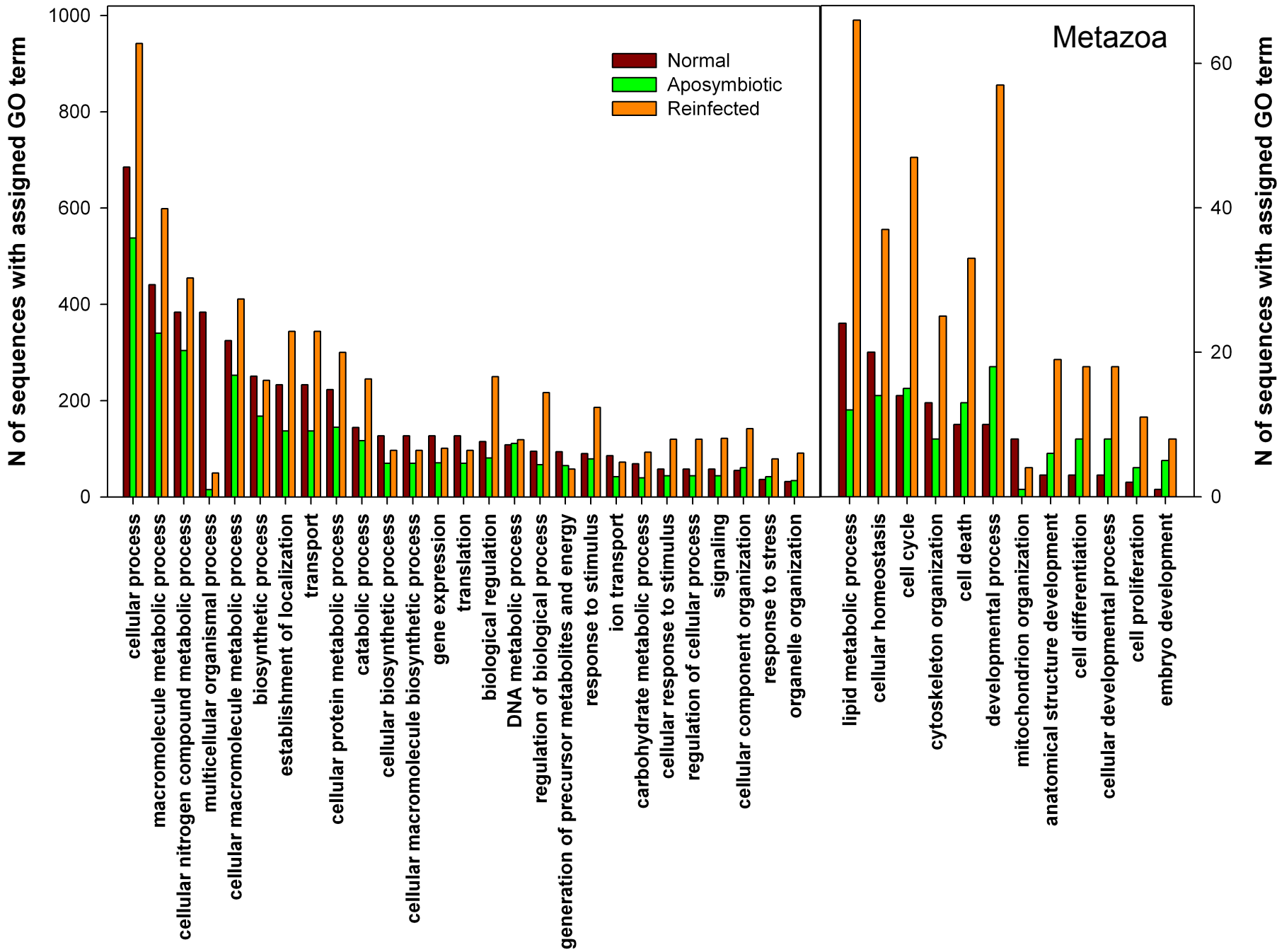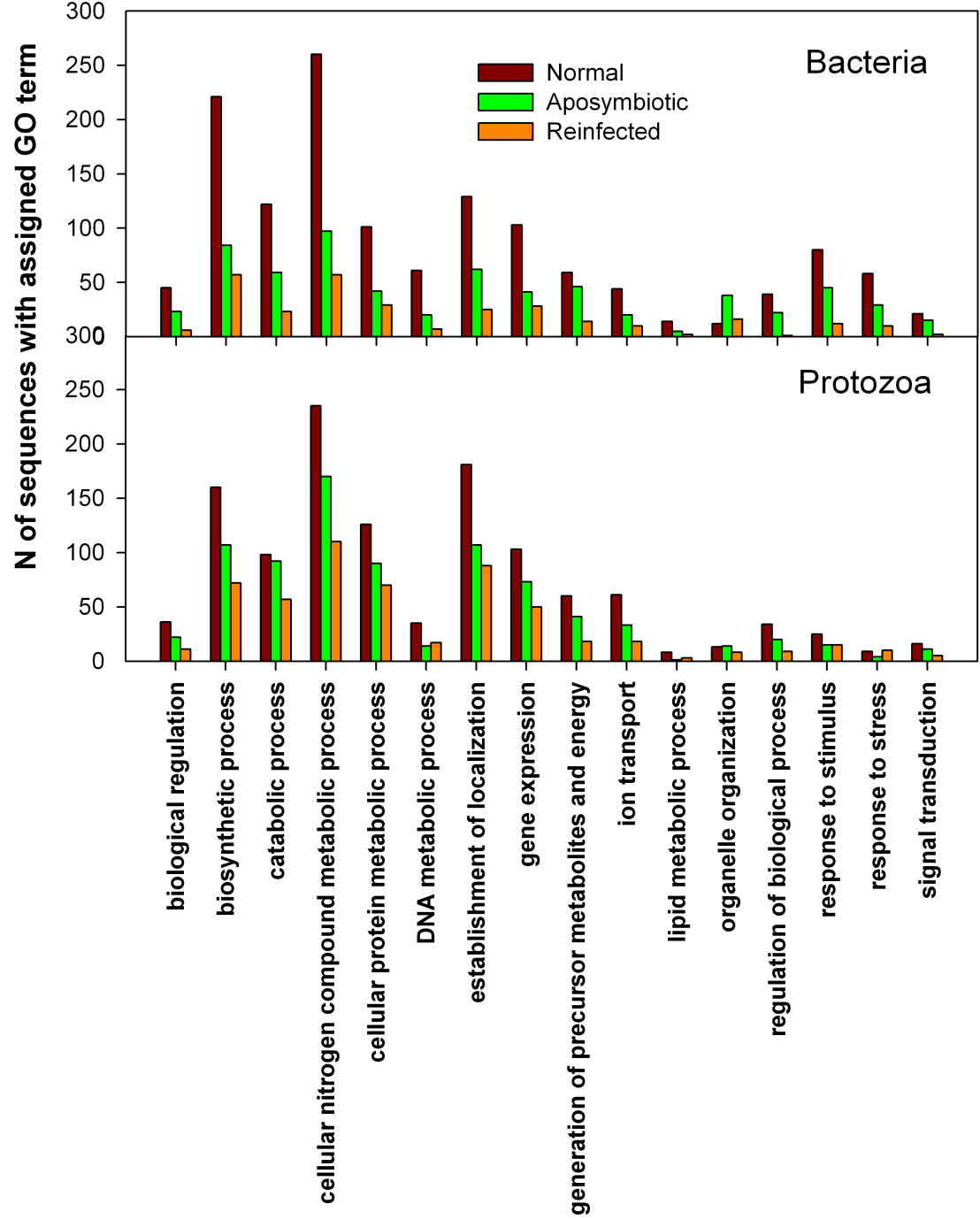

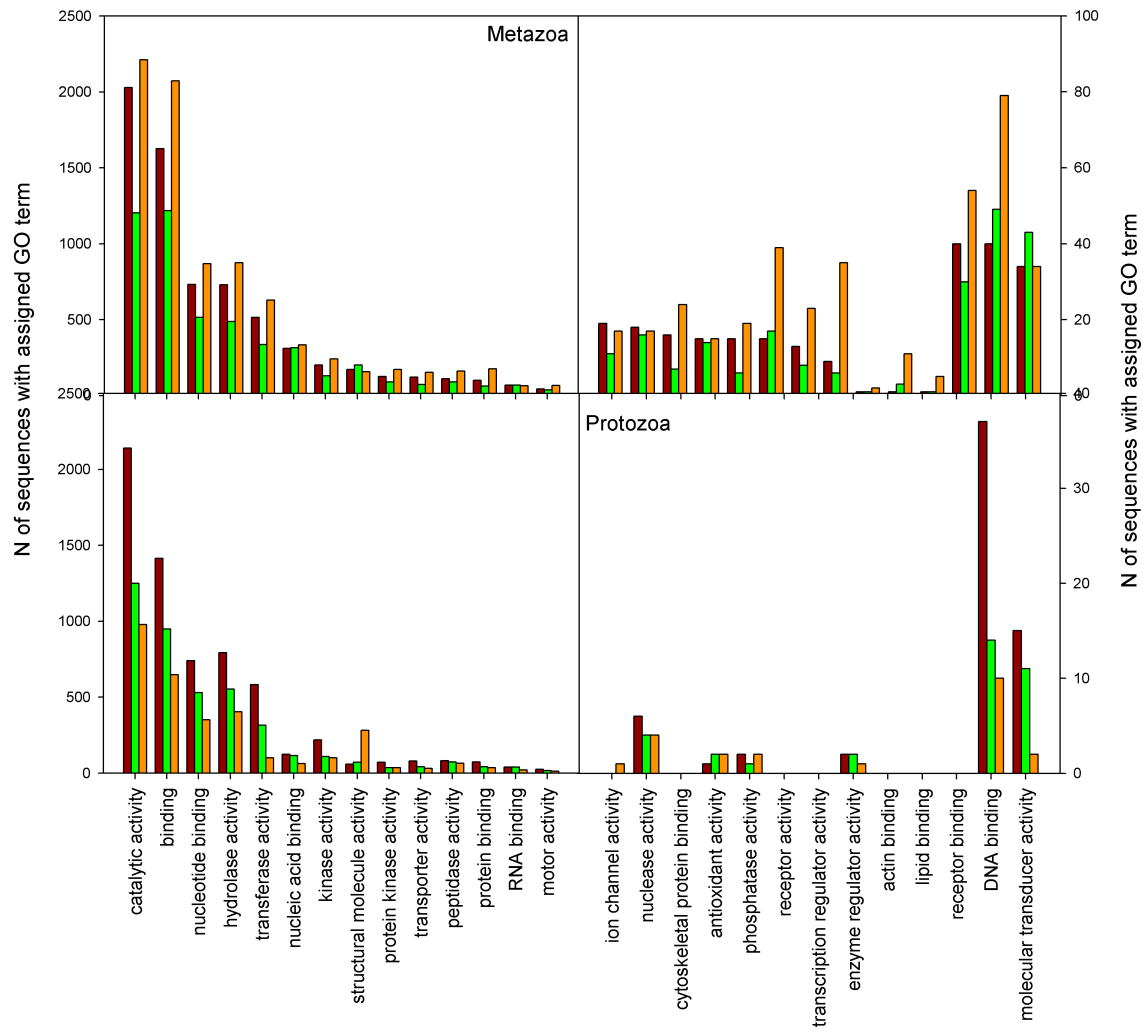

## Molecular Function

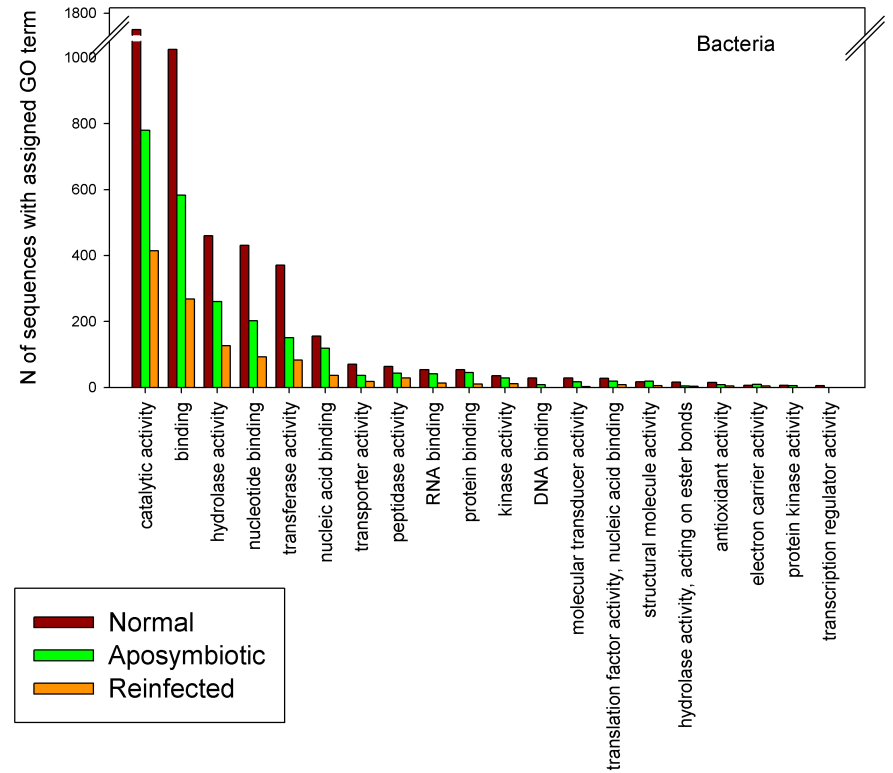

Cellular component

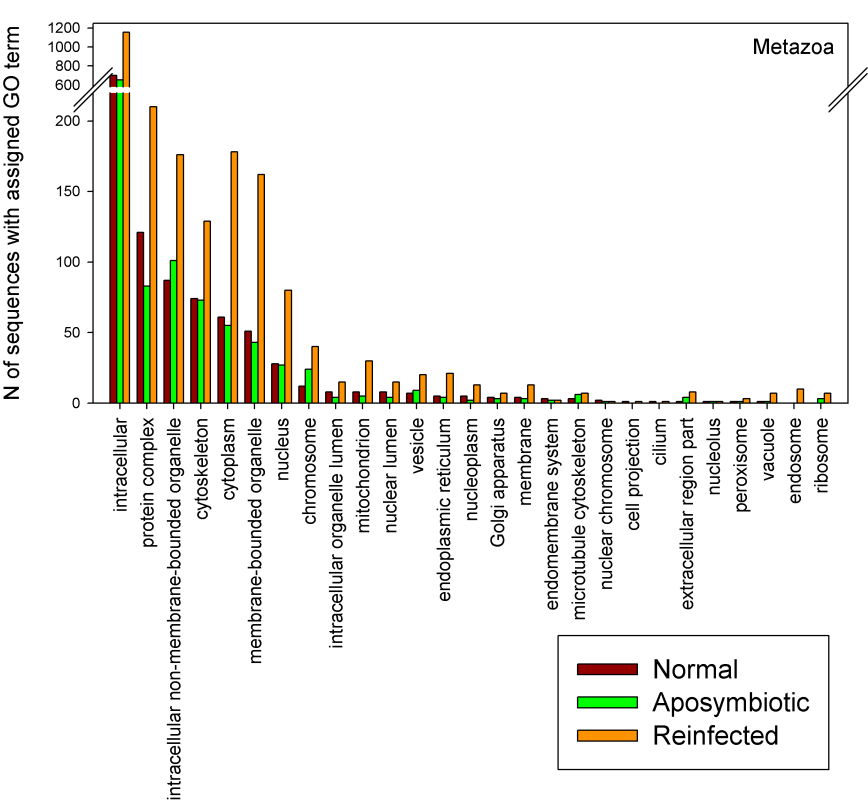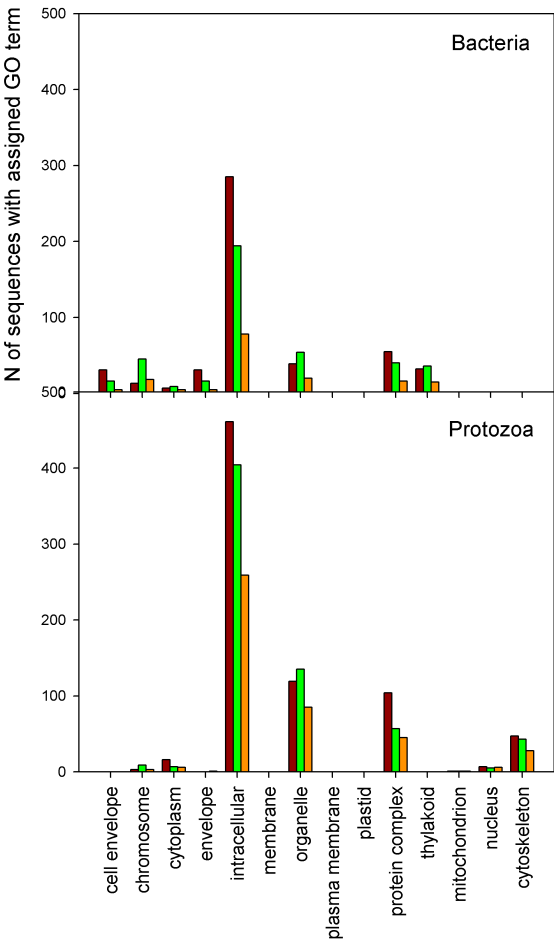

Supplement: Supplementary file 3 — Additional file 3: Figure S2: A. GO term assignment for the biological process category in each transcriptomic dataset (“normal”, “aposymbiotic”, and “reinfected” treatments) when using the databases Metazoa, Bacteria, and Protozoa (subselections of the nr database from NCBI). B. GO term assignment for the molecular function category in each transcriptomic dataset (“normal”, “aposymbiotic”, and “reinfected” treatments) when using the databases Metazoa, Bacteria, and Protozoa (subselections of the nr database from NCBI). C. GO term assignment for the cellular component category in each transcriptomic dataset (“normal”, “aposymbiotic”, and “reinfected” treatments) when using the databases Metazoa, Bacteria, and Protozoa (subselections of the nr database from NCBI). (ZIP 2 MB) [file 12864_2013_6178_MOESM3_ESM.zip › 1303700591046817_add2/1303700591046817_add2a.pdf]

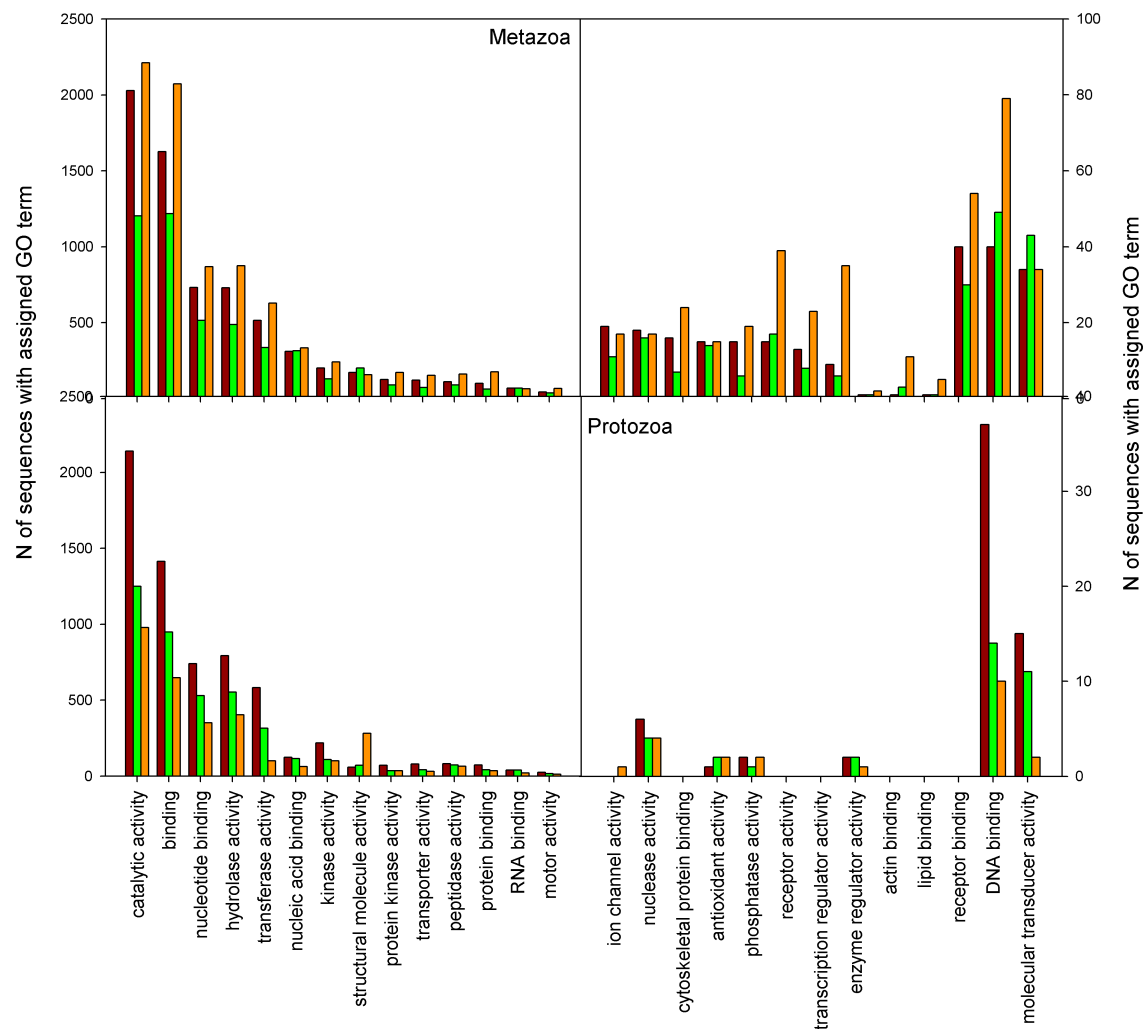

## Molecular Function

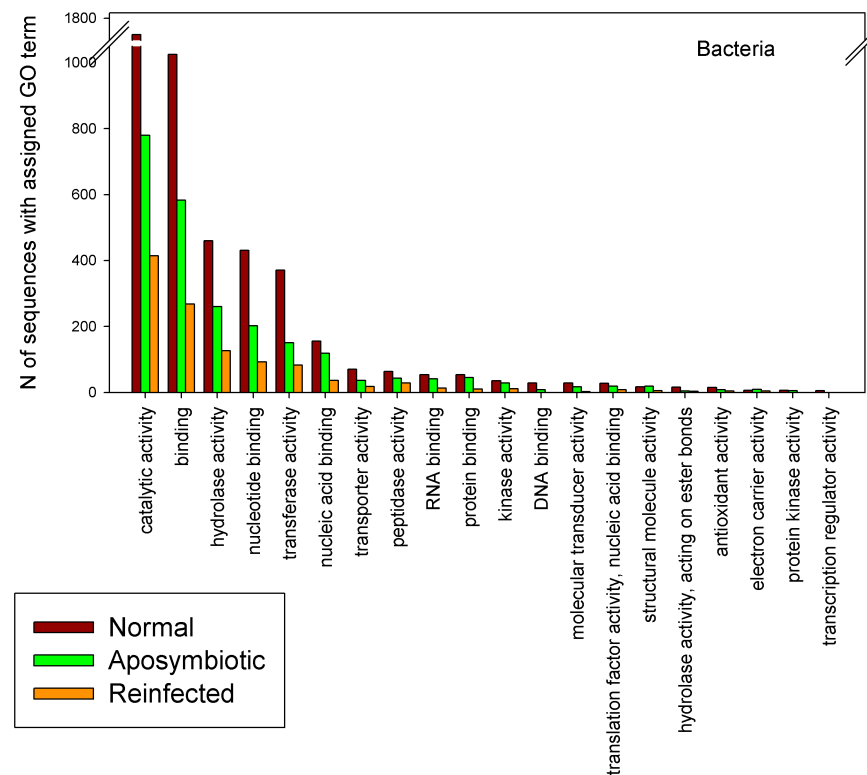

Supplement: Supplementary file 3 — Additional file 3: Figure S2: A. GO term assignment for the biological process category in each transcriptomic dataset (“normal”, “aposymbiotic”, and “reinfected” treatments) when using the databases Metazoa, Bacteria, and Protozoa (subselections of the nr database from NCBI). B. GO term assignment for the molecular function category in each transcriptomic dataset (“normal”, “aposymbiotic”, and “reinfected” treatments) when using the databases Metazoa, Bacteria, and Protozoa (subselections of the nr database from NCBI). C. GO term assignment for the cellular component category in each transcriptomic dataset (“normal”, “aposymbiotic”, and “reinfected” treatments) when using the databases Metazoa, Bacteria, and Protozoa (subselections of the nr database from NCBI). (ZIP 2 MB) [file 12864_2013_6178_MOESM3_ESM.zip › 1303700591046817_add2/1303700591046817_add2b.pdf]

Cellular component

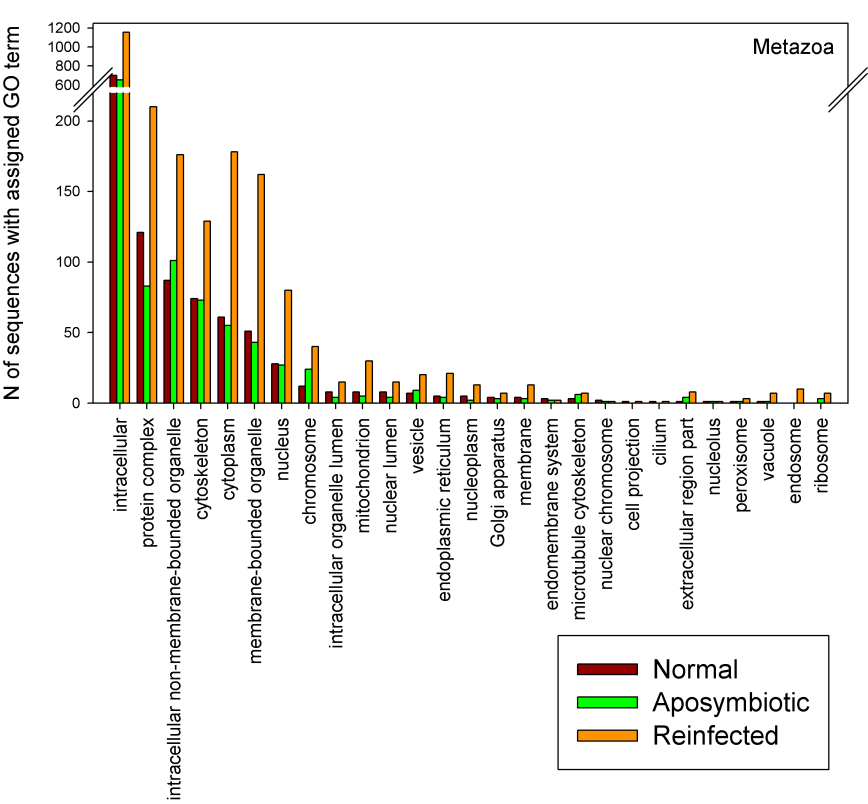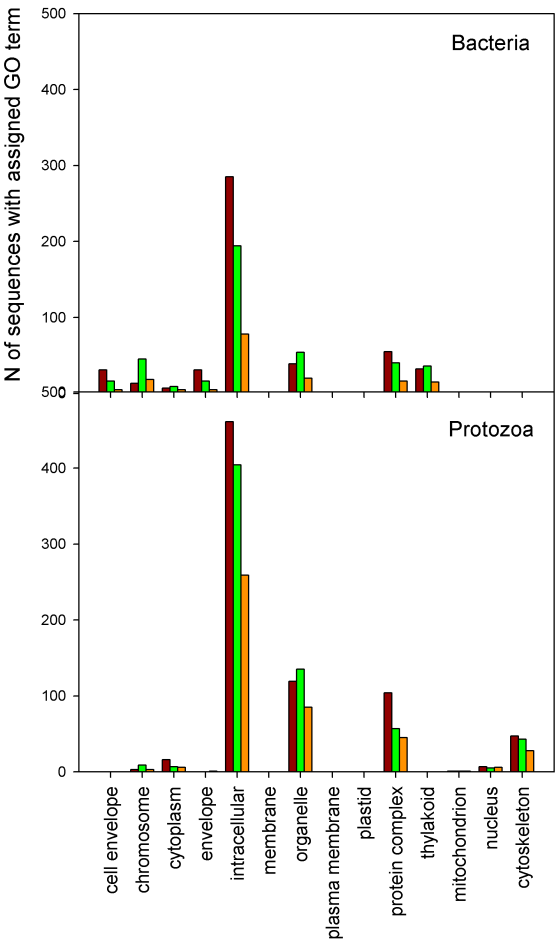

Supplement: Supplementary file 3 — Additional file 3: Figure S2: A. GO term assignment for the biological process category in each transcriptomic dataset (“normal”, “aposymbiotic”, and “reinfected” treatments) when using the databases Metazoa, Bacteria, and Protozoa (subselections of the nr database from NCBI). B. GO term assignment for the molecular function category in each transcriptomic dataset (“normal”, “aposymbiotic”, and “reinfected” treatments) when using the databases Metazoa, Bacteria, and Protozoa (subselections of the nr database from NCBI). C. GO term assignment for the cellular component category in each transcriptomic dataset (“normal”, “aposymbiotic”, and “reinfected” treatments) when using the databases Metazoa, Bacteria, and Protozoa (subselections of the nr database from NCBI). (ZIP 2 MB) [file 12864_2013_6178_MOESM3_ESM.zip › 1303700591046817_add2/1303700591046817_add2c.pdf]

**Total experiment**  
**(292.182 contigs)**

**N      A      R**

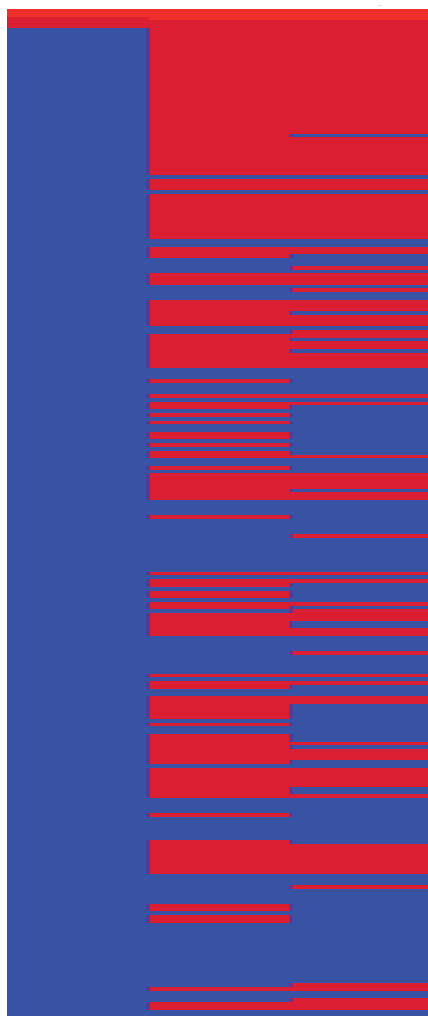

**Contigs over 1000 bp**  
**(15.636 contigs)**

**N      A      R**

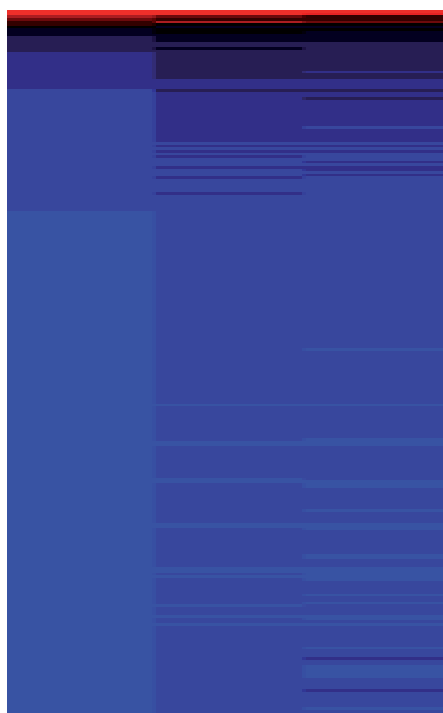

**Selection Difference = 2**  
**(13.773 contigs)**

**N      A      R**

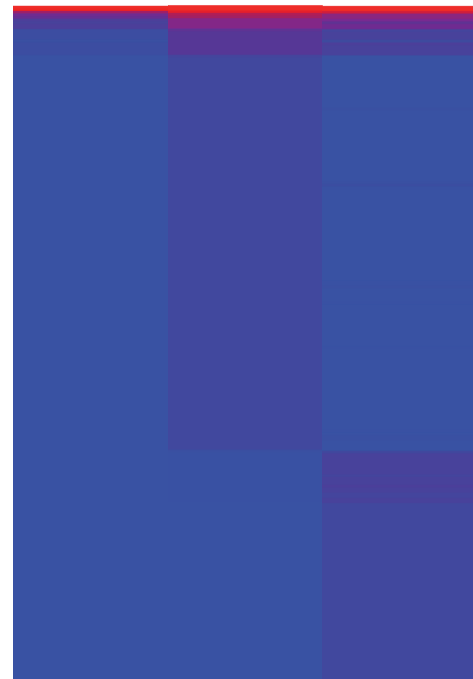

Supplement: Supplementary file 5 — Additional file 5: Figure S3: Heat maps showing the expression levels of the total dataset, a subselection of contigs over 1,000 bp, and a subselection of contigs showing a difference of 2. (PDF 354 KB) [file 12864_2013_6178_MOESM5_ESM.pdf]
