# Supplementary material for: Transcriptomic analysis of differential host gene expression upon uptake of symbionts: a case study with Symbiodinium and the major bioeroding sponge Cliona varians
Source: BMC Genomics. 2014 May 16;15(1):376. doi: 10.1186/1471-2164-15-376 (PMC4144087; doi:10.1186/1471-2164-15-376)
Supplement: Supplementary file 2 — Additional file 2: Table S1: Contigs from all sources (e.g., metazoan, bacterial, and protozoan) that showed significantly different expression values in the “normal” vs. “aposymbiotic” and “aposymbiotic” vs. “reinfected” treatment comparisons (from Figure 4). Adjusted p values and protein names are shown. (PDF 83 KB) [file 12864_2013_6178_MOESM2_ESM.pdf]

IPPLEMENTAL TABLE 1

Normal vs Aposymbiotic

Increased in Normal

Contig name	Protein name	Padj	Blast vs
Contig_273440	V-type ATP synthase subunit F	0.012472931	Bacteria
Contig_61915	peptidylprolyl isomerase	0.012472931	
Contig_55287	nadh dehydrogenase subunit 5	0.01775091	
Contig_55333	collagen-like surface protein	0.024090016	
Contig_158889	aminotransferase	0.0280811	
Contig_220009	polymerase	0.038073654	
Contig_55166	chorismate mutase	0.038073654	
Contig_55141	DNA-directed RNA polymerase subunit beta'	0.040932326	
Contig_204175	phasin	0.044018908	
Contig_55083	diphosphate--fructose-6-phosphate 1-phosphotransferase	0.048902651	
Contig_59117	proteinase inhibitor	0.051695464	
Contig_73310	Porin	0.051695464	
Contig_51777	astacin	0.052732041	
Contig_72576	cell-division initiation protein	0.076345247	
Contig_261711	cytochrome oxidase II	0.012472931	Metazoa
Contig_264833	hemicentin-2	0.012472931	
Contig_59937	fibronectin	0.012472931	
Contig_199414	cytochrome oxidase I	0.016723914	
Contig_72552	basement membrane-specific heparan sulfate proteoglycan core protein-like	0.01775091	
Contig_72648	TNF receptor-associated factor 3-like	0.024090016	
Contig_60858	deleted in malignant brain tumors 1 protein-like, partial	0.0280811	
Contig_204347	lysyl-tRNA synthetase-like	0.035259701	
Contig_199206	RING finger protein	0.03805432	
Contig_72489	astacin	0.03805432	
Contig_157422	ALK tyrosine kinase receptor-like	0.03805432	
Contig_199383	TNF receptor-associated factor 3-like	0.051067272	
Contig_63245	sporulation-specific protein 15-like	0.061312247	
Contig_261743	X-linked retinitis pigmentosa GTPase regulator-interacting protein 1	0.063559639	
Contig_73070	Ig heavy chain V-II region WAH	0.064679662	
Contig_55539	tetratricopeptide repeat protein 28-like isoform X3	0.065062182	
Contig_200816	collagen alpha-1(I) chain	0.071566318	
Contig_263087	aggregation factor protein 3, form D	0.076345247	
Contig_55107	TPR repeat-containing protein, variant	0.076345247	

Increased in Aposymbiotic

Contig name	Protein name	Padj	Blast vs
Contig_106477	metallopeptidase	0.071866078	Bacteria
Contig_156986	O-acetylhomoserine aminocarboxypropyltransferase/cysteine synthase	0.054505458	
Contig_157706	crinkler family protein	0.070687793	
Contig_201312	lipopolysaccharide biosynthesis protein	0.016723914	
Contig_238382	DNA topoisomerase IV subunit A	0.016723914	
Contig_262191	malonate decarboxylase subunit delta	0.054967488	
Contig_269005	sodium/hydrogen exchanger	0.012662663	
Contig_59342	membrane protein	0.012472931	
Contig_200895	methyl accepting chemotaxis protein	0.012472931	
Contig_59396	gut cathepsin l-like cysteine protease	0.023378011	
Contig_59497	DEAD/DEAH box helicase	0.0402445	
Contig_61555	cell surface A33 antigen	0.052202094	
Contig_59339	tpr repeat-containing protein	0.026333566	Cyanobacteria
Contig_50894	dynamin	0.023378011	Fungi
Contig_156555	DBH-like monooxygenase protein 1	0.096926504	Metazoa
Contig_156675	ATPase	0.051695464	
Contig_156684	cartilage intermediate layer protein	0.048902651	
Contig_157717	sulfide:quinone oxidoreductase, mitochondrial-like	0.023378011	
Contig_200019	death domain-containing protein	0.01775091	
Contig_200382	GTPase IMAP family member 7-like isoform X4	0.012472931	
Contig_200388	Ras-related protein	0.023378011	
Contig_202394	zinc finger MYM-type protein 1-like	0.013414074	
Contig_208315	titin	0.013301206	
Contig_208383	GTPase IMAP family member 4 isoform X1	0.012472931	
Contig_211234	hemicentin	0.012472931	
Contig_262244	host cell factor 2-like	0.013301206	
Contig_263151	zinc finger MYM-type protein 1-like	0.012472931	
Contig_264371	short-chain collagen C4-like	0.035259701	
Contig_268330	protein sidekick-1	0.091240263	
Contig_268997	immunoglobulin-like and fibronectin type III domain-containing protein 1 isoform X2	0.083728603	
Contig_271928	collagen alpha-1(XII) chain-like	0.012472931	
Contig_276279	GTPase IMAP family member 4 isoform a	0.020568358	
Contig_281237	ephryn type A-receptor	0.01775091	
Contig_56775	interferon-induced very large gtpase 1-like	0.052202094	
Contig_57113	phenylalanyl hydroxylase	0.083728603	
Contig_58260	phenylalanyl-tRNA synthetase beta chain	0.01775091	
Contig_59159	aggregation factor protein 3	0.024025106	
Contig_59391	cre-nas-21 protein	0.048902651	
Contig_59411	fibrillin-1- partial	0.012472931	
Contig_59415	poly [ADP-ribose] polymerase 14	0.020919639	
Contig_59431	sideroflexin-2	0.044018908	
Contig_61716	collagen alpha-1(XII) chain-like	0.012472931	
Contig_79428	phenylalanine--tRNA ligase alpha subunit-like	0.034521852	
Contig_95189	bifunctional aminoacyl-trna synthetase	0.012472931	
Contig_160769	kinesin heavy-chain like protein	0.022995743	Protozoa
Contig_268963	E1 protein	0.081709943	Virus

Aposymbiotic vs. Reinfected

Increased in Aposymbiotic

Contig name	Protein name	Padj	Blast vs
Contig_263256	valine decarboxylase	0.037757567	Bacteria
Contig_117675	anykirin	0.028697348	
Contig_161887	polymorphic outer membrane protein	0.017569095	
Contig_269005	sodium/hydrogen exchanger	0.007252336	
Contig_59497	type iii restriction protein res subunit	0.053137863	
Contig_208383	gtpase imap family member 4	0.00023642	Metazoa
Contig_200382	aig1 domain-containing protein	0.000956975	
Contig_59411	fibrillin-1- partial	0.001201322	
Contig_95189	bifunctional aminoacyl-trna synthetase	0.001361404	
Contig_271928	collagen alpha-1 chain-like	0.00191647	
Contig_270097	hydrocephalus-inducing protein homolog	0.002657635	
Contig_156716	PREDICTED: hypothetical protein LOC100640736*	0.005679118	
Contig_59342	membrane protein	0.0063882	
Contig_221198	PREDICTED: hypothetical protein LOC100631580*	0.007252336	
Contig_194451	metallothionein	0.010809295	
Contig_58260	phenylalanyl-trna synthetase beta chain	0.016979231	
Contig_281237	ephrin type-A receptor 5 isoform X1	0.017569095	
Contig_200019	PREDICTED: hypothetical protein LOC100639474*	0.017569095	
Contig_106607	dynein heavy chain 6 axonemal-like	0.018033908	
Contig_276279	GTPase IMAP family member	0.020364059	
Contig_59415	poly ADP ribose polymerase 14-like	0.02116981	
Contig_160769	kinesin heavy-chain-like protein	0.023632164	
Contig_59396	cathepsin l-like cysteine protease	0.025254867	
Contig_59159	aggregation factor protein 3	0.027240666	
Contig_262191	integrase core domain protein	0.030901343	
Contig_79428	phenylalanine--trna ligase alpha subunit isoform 1	0.041888037	
Contig_221381	short-chain collagen c4-like	0.041888037	
Contig_156555	dbh-like monooxygenase protein 1 homolog	0.051920102	
Contig_216885	bcl-2-like protein 1-like	0.052603989	
Contig_156696	cell surface A33 antigen	0.056916824	
Contig_222064	plekstrin homology-like domain family B member 2-like	0.05765085	
Contig_277901	methylmalonyl-CoA carboxyltransferase	0.05795929	
Contig_59431	sideroflexin 2	0.05795929	
Contig_59391	cre-nas-21 protein	0.064815123	
Contig_236109	tnf receptor-associated factor 6	0.064815123	
Contig_235499	bhp1 protein	0.06699101	
Contig_157717	sulfide:quinone mitochondrial	0.067861887	
Contig_230629	methylmalonic aciduria and homocystinuria type d mitochondrial-like	0.06872428	
Contig_221777	tetraspanin-3-like isoform 2	0.069472619	
Contig_56775	interferon-induced very large gtpase 1-like	0.072813671	
Contig_69803	probable inactive purple acid phosphatase 27-like	0.072813671	
Contig_219193	PREDICTED: uncharacterized protein K02A2.6-like	0.085088634	
Contig_129806	Eph receptor tyrosine kinase	0.085986807	
Contig_156525	membrane protein	0.088260571	
Contig_221292	selenocysteine lyase	0.088260571	
Contig_265659	Anoctamin	0.094340998	
Contig_204298	tolloid-like protein 1	0.094340998	
Contig_157706	crinkler family	0.096901234	Fungi

Increased in Reinfected

Contig name	Protein name	Padj	Blast vs
Contig_210604	glutamyl endopeptidase	0.000409321	Bacteria
Contig_210730	glutamyl endopeptidase	0.000480181	
Contig_210563	DNA-polymerase	0.004304005	
Contig_159508	collagen-like cell surface-anchored protein SclH	0.017569095	
Contig_262626	conserved protein	0.018497501	
Contig_262635	lipoprotein	0.026591576	
Contig_51363	viral a-type inclusion protein	0.030901343	
Contig_270979	ATP-dependent helicase	0.031414813	
Contig_99090	beta gamma crystallin	0.072813671	
Contig_212496	perforin-like protein	0.078179642	
Contig_260915	hemicentin 2	0.000448262	Metazoa
Contig_210598	sarcoplasmic calcium-binding	0.000448262	
Contig_219184	fibrinogen c domain-containing protein 1-a-like	0.000755112	
Contig_210698	low quality protein: titin	0.001338735	
Contig_61652	hypothetical protein BRAFLDRAFT_78705	0.001500212	
Contig_105978	modified aequorin	0.001598761	
Contig_61670	2-5a oligoadenylate synthetase	0.002195287	
Contig_211183	hypothetical protein CGI_10021602	0.002881968	
Contig_262832	conserved hypothetical protein	0.003376712	
Contig_61779	peptidase m12a astacin	0.003982725	
Contig_156876	proprotein convertase subtilisin kexin type 9-like	0.004751185	
Contig_51058	scribble	0.011009614	
Contig_62126	heme-binding protein 2-like	0.020364059	
Contig_51057	interferon-induced very large gtpase 1-like	0.024470435	
Contig_59765	protein g7c-like	0.033483902	
Contig_271027	fibrinogen-like protein a	0.035792456	
Contig_176284	calcium binding protein	0.05795929	
Contig_263232	synaptophysin b	0.059542099	
Contig_198461	short-chain collagen c4-like	0.06359224	
Contig_263087	aggregation factor protein 3	0.078179642	
Contig_61631	protein isoform a	0.08948477	
Contig_261263	proprotein convertase subtilisin kexin type 9-like	0.094340998	
Contig_161256	sarcoplasmic calcium-binding	0.094340998	
