# Supplementary material for: Transcriptomic analysis of differential host gene expression upon uptake of symbionts: a case study with Symbiodinium and the major bioeroding sponge Cliona varians
Source: BMC Genomics. 2014 May 16;15(1):376. doi: 10.1186/1471-2164-15-376 (PMC4144087; doi:10.1186/1471-2164-15-376)
Supplement: Supplementary file 4 — Additional file 4: Table S4: Metazoan contigs that showed significantly different expression values in the “normal” vs. “aposymbiotic” and “aposymbiotic” vs. “reinfected” treatment comparisons (from Figure 4). Adjusted p values and protein names are shown. (PDF 48 KB) [file 12864_2013_6178_MOESM4_ESM.pdf]

TABLE 2

Normal vs Aposymbiotic					
Increased in Aposymbiotic			Increased in Normal		
Contig name	Protein name	Padj	Contig name	Protein name	Padj
Contig_59159	aggregation factor protein 3	0.02402511	Contig_263087	aggregation factor protein 3, form D	0.07634525
Contig_200382	aig1 domain-containing protein	0.01247293	Contig_157422	ALK tyrosine kinase receptor-like	0.03805432
Contig_156675	ATPase	0.05169546	Contig_72489	astacin	0.03805432
Contig_95189	bifunctional aminoacyl-trna synthetase	0.01247293	Contig_72552	basement membrane-specific heparan sulfate proteoglycan core protein-like	0.01775091
Contig_156684	cartilage intermediate layer protein	0.04890265	Contig_200816	collagen alpha-1(I) chain	0.07156632
Contig_271928	collagen alpha-1(XII) chain-like	0.01247293	Contig_199414	cytochrome oxidase I	0.01672391
Contig_61716	collagen alpha-1(XII) chain-like	0.01247293	Contig_261711	cytochrome oxidase II	0.01247293
Contig_59391	cre-nas-21 protein	0.04890265	Contig_60858	deleted in malignant brain tumors 1 protein-like, partial	0.0280811
Contig_156555	DBH-like monooxygenase protein 1	0.0969265	Contig_59937	fibronectin	0.01247293
Contig_200019	death domain-containing protein	0.01775091	Contig_264833	hemicentin-2	0.01247293
Contig_281237	ephryn type A-receptor	0.01775091	Contig_73070	Ig heavy chain V-II region WAH	0.06467966
Contig_59411	fibrillin-1- partial	0.01247293	Contig_204347	lysyl-tRNA synthetase-like	0.0352597
Contig_276279	GTPase IMAP family member 4 isoform a	0.02056836	Contig_199206	RING finger protein	0.03805432
Contig_208383	GTPase IMAP family member 4 isoform X1	0.01247293	Contig_55539	tetratricopeptide repeat protein 28-like isoform X3	0.06506218
Contig_211234	hemicentin	0.01247293	Contig_72648	TNF receptor-associated factor 3-like	0.02409002
Contig_262244	host cell factor 2-like	0.01330121	Contig_199383	TNF receptor-associated factor 3-like	0.05106727
Contig_268997	immunoglobulin-like and fibronectin type III domain-containing protein 1 isof(	0.0837286	Contig_55107	TPR repeat-containing protein, variant	0.07634525
Contig_56775	interferon-induced very large gtpase 1-like	0.05220209	Contig_261743	X-linked retinitis pigmentosa GTPase regulator-interacting protein 1	0.06355964
Contig_79428	phenylalanine--tRNA ligase alpha subunit-like	0.03452185			
Contig_57113	phenylalanyl hydroxylase	0.0837286			
Contig_58260	phenylalanyl-tRNA synthetase beta chain	0.01775091			
Contig_59415	poly [ADP-ribose] polymerase 14	0.02091964			
Contig_268330	protein sidekick-1	0.09124026			
Contig_200388	Ras-related protein	0.02337801			
Contig_264371	short-chain collagen C4-like	0.0352597			
Contig_59431	sideroflexin-2	0.04401891			
Contig_157717	sulfide:quinone oxidoreductase, mitochondrial-like	0.02337801			
Contig_208315	titin	0.01330121			
Contig_202394	zinc finger MYM-type protein 1-like	0.01341407			
Contig_263151	zinc finger MYM-type protein 1-like	0.01247293			

Aposymbiotic vs Reinfected					
Increased in Reinfected			Increased in Aposymbiotic		
Contig name	Protein name	Padj	Contig name	Protein name	Padj
Contig_51057	interferon-induced very large gtpase 1-like	0.02447044	Contig_208383	gtpase imap family member 4	0.00023642
Contig_263232	synaptophysin b	0.0595421	Contig_200382	aig1 domain-containing protein	0.00095698
Contig_61670	2-5a oligoadenylate synthetase	0.00219529	Contig_59411	fibrillin-1- partial	0.00120132
Contig_263087	aggregation factor protein 3	0.07817964	Contig_95189	bifunctional aminoacyl-trna synthetase	0.0013614
Contig_176284	calcium binding protein	0.05795929	Contig_271928	collagen alpha-1 chain-like	0.00191647
Contig_262832	conserved hypothetical protein	0.00337671	Contig_270097	hydrocephalus-inducing protein homolog	0.00265764
Contig_219184	fibrinogen c domain-containing protein 1-a-like	0.00075511	Contig_156716	PREDICTED: hypothetical protein LOC100640736*	0.00567912
Contig_271027	fibrinogen-like protein a	0.03579246	Contig_59342	membrane protein	0.0063882
Contig_62126	heme-binding protein 2-like	0.02036406	Contig_221198	PREDICTED: hypothetical protein LOC100631580*	0.00725234
Contig_260915	hemicentin 2	0.00044826	Contig_194451	metallothionein	0.0108093
Contig_61652	hypothetical protein BRAFLDRAFT_78705	0.00150021	Contig_58260	phenylalanyl-trna synthetase beta chain	0.01697923
Contig_211183	hypothetical protein CGI_10021602	0.00288197	Contig_281237	ephrin type-A receptor 5 isoform X1	0.0175691
Contig_210698	low quality protein: titin	0.00133874	Contig_200019	PREDICTED: hypothetical protein LOC100639474*	0.0175691
Contig_105978	modified aequorin	0.00159876	Contig_106607	dynein heavy chain 6 axonemal-like	0.01803391
Contig_61779	peptidase m12a astacin	0.00398273	Contig_276279	GTPase IMAP family member	0.02036406
Contig_156876	proprotein convertase subtilisin kexin type 9-like	0.00475119	Contig_59415	poly ADP ribose polymerase 14-like	0.02116981
Contig_261263	proprotein convertase subtilisin kexin type 9-like	0.094341	Contig_160769	kinesin heavy-chain-like protein	0.02363216
Contig_59765	protein g7c-like	0.0334839	Contig_59396	cathepsin l-like cysteine protease	0.02525487
Contig_61631	protein isoform a	0.08948477	Contig_59159	aggregation factor protein 3	0.02724067
Contig_210598	sarcoplasmic calcium-binding	0.00044826	Contig_262191	integrase core domain protein	0.03090134
Contig_161256	sarcoplasmic calcium-binding	0.094341	Contig_79428	phenylalanine--trna ligase alpha subunit isoform 1	0.04188804
Contig_51058	scribble	0.01100961	Contig_221381	short-chain collagen c4-like	0.04188804
Contig_198461	short-chain collagen c4-like	0.06359224	Contig_156555	dbh-like monooxygenase protein 1 homolog	0.0519201
			Contig_216885	bcl-2-like protein 1-like	0.05260399
			Contig_156696	cell surface A33 antigen	0.05691682
			Contig_222064	plekstrin homology-like domain family B member 2-like	0.05765085
			Contig_277901	methylmalonyl-CoA carboxyltransferase	0.05795929
			Contig_59431	sideroflexin 2	0.05795929
			Contig_59391	cre-nas-21 protein	0.06481512
			Contig_236109	tnf receptor-associated factor 6	0.06481512
			Contig_235499	bhp1 protein	0.06699101
			Contig_157717	sulfide:quinone mitochondrial	0.06786189
			Contig_230629	methylmalonic aciduria and homocystinuria type d mitochondrial-like	0.06872428
			Contig_221777	tetraspanin-3-like isoform 2	0.06947262
			Contig_56775	interferon-induced very large gtpase 1-like	0.07281367
			Contig_69803	probable inactive purple acid phosphatase 27-like	0.07281367
			Contig_219193	PREDICTED: uncharacterized protein K02A2.6-like	0.08508863
			Contig_129806	Eph receptor tyrosine kinase	0.08598681
			Contig_156525	membrane protein	0.08826057
			Contig_221292	selenocysteine lyase	0.08826057
			Contig_265659	Anoctamin	0.094341
			Contig_204298	tolloid-like protein 1	0.094341
