# Supplementary material for: Transcriptomic analysis of differential host gene expression upon uptake of symbionts: a case study with Symbiodinium and the major bioeroding sponge Cliona varians
Source: BMC Genomics. 2014 May 16;15(1):376. doi: 10.1186/1471-2164-15-376 (PMC4144087; doi:10.1186/1471-2164-15-376)
Supplement: Supplementary file 6 — Additional file 6: Table S2: List of the contig assignments for each of the genes represented in Figures 7 and 8. (PDF 51 KB) [file 12864_2013_6178_MOESM6_ESM.pdf]

Supplemental Table 2

ENDOSOME, LYSOSOME, PHAGOSOME DATA (From Figure 7)

Most significant BLAST result	Contig ID	Reinf/Apo
protein tyrosine kinase	51123	32.9
receptor-type tyrosine-protein phosphatase f isoform 2 precursor	63341	10.1
a chain crystal structure of insulin-like growth factor 1 receptor (igf-1r-wt)	66172	10.1
copper-transporting atpase 1	202378	6.3
solute carrier family 15 member 3	71004	5.1
beta-glucuronidase-like	65668	5.1
phosphatidylinositol 3-kinase catalytic subunit type 3	53024	4.3
sialin-like	156883	4.0
BAI1-associated protein 3-like	64657	3.9
vesicle-associated membrane synaptobrevin 7b	63664	3.7
ankyrin repeat domain-containing protein 27	52683	3.4
grip1-associated protein 1	262908	3.3
proteasome-associated protein ecm29 homolog	62987	3.2
protein tyrosine receptor f	57138	3.2
glucosylceramidase	200990	3.1
two pore calcium channel protein 1A	64058	3.0
wd repeat-containing protein 48	161276	2.8
bromodomain and wd repeat-containing protein 3	53544	2.8
phospholipase b-like 2-like	63504	2.6
lysosomal alpha-mannosidase	261092	2.6
ephrin type-a receptor 4-like	51948	2.6
tnf receptor-associated factor 3-like	201416	2.4
cathepsin b	202630	2.4
efflux abc transporter permease atp-binding protein	63735	2.3
h(+) cl(-) exchange transporter 3 isoform b	198883	2.3
tnf receptor-associated factor 2/3-like A	260971	2.3
e3 ubiquitin-protein ligase mib2-like A	156452	2.2
n-acylethanolamine-hydrolyzing acid amidase-like	198571	2.2
run and fyve domain containing 2	63441	2.2
ras-related protein rab-22a	201547	2.2
sushi, von Willebrand factor type A, EGF, and pentraxin domain-containing protein 1-like	209184	2.1
charged multivesicular body protein 1b	62056	2.1
t-complex protein 1 subunit alpha-like	198605	2.1
tnf receptor-associated factor 2/3-like B	260911	2.1
rab family GTPase	61946	2.1
mit domain-containing protein 1	202199	2.1
cap-gly domain-containing linker protein 1	200503	2.0
e3 ubiquitin-protein ligase mib2-like B	62707	2.0
heparan-alpha-glucosaminide n-acetyltransferase-like	199277	16.6
ubiquitin isoform cra_a	54372	9.2
deleted in malignant brain tumors 1 protein-like E	156484	8.6
phosphotidylinositol phosphatase pt prq-like	261661	8.5
chloride transport protein 6B	266437	6.7
chloride transport protein 6A	263101	4.8
prolow-density lipoprotein receptor-related protein 1	156836	4.7
run domain beclin-1 interacting and cystein-rich	264124	4.7
zinc finger fyve domain-containing protein 16	199661	4.6
solute carrier family 15 member 4	54904	4.0
receptor-type tyrosine-protein phosphatase	199159	3.9
niemann-pick c1 protein C	161416	3.8
1-phosphatidylinositol-3-phosphate 5-kinase	264623	3.7
serine/threonine-protein kinase mTOR-like	64141	3.6
polyphosphoinositide phosphatase	62699	3.6
mitogen-activated protein kinase kinase kinase kinase 4 isoform 4	51365	3.5
scy1-like protein 2	156907	3.5
deleted in malignant brain tumors 1 protein-like D	262647	3.4
niemann-pick c1 protein B	62495	3.4
poly(adp-ribose) glycohydrolase-like	52192	3.3
sid1 transmembrane family member 1	66743	3.3
ATP-binding sub-family a member 2	203188	3.1
e3 ubiquitin-protein ligase mib2-like C	53120	3.1
receptor-type tyrosine-protein phosphatase s	204010	3.1
ubiquitin c	68188	3.0
probable serine threonine-protein kinase	66020	3.0
ATP-binding cassette sub-family b member 9	201838	2.9
niemann-pick c1 protein A	263205	2.8
nischarin	51310	2.7
dedicator of cytokinesis protein 2-like	65139	2.6
deleted in malignant brain tumors 1 protein-like C	201276	2.6
two pore calcium channel protein 1 B	213082	2.6
lipopolysaccharide-responsive and beige-like anchor protein	51074	2.5
zinc fyve domain containing 20	202127	2.5
phospholipase d2	99536	2.4
deleted in malignant brain tumors 1 protein-like B	165194	2.4
GTPase-activating protein and vps9 domain-containing protein 1	51754	2.4
huntingtin	51763	2.2
deleted in malignant brain tumors 1 protein-like A	201701	2.1
sans isoform a	201809	2.0
neuropathy target esterase	52095	2.0

CELL DIVISION DATA (From Figure 8A)

Most significant BLAST result	Contig ID	Reinf/Apo
bcl-2 homology protein	166238	17.9
Serine/threonine-protein kinase LMTK1	66172	10.1
mitogen-activated protein kinase kinase kinase 3-like	156942	5.9
cell division cycle protein 23 homolog	261073	5.7
centromere protein j	263874	5.7
phosphatidylinositol 3-kinase catalytic subunit type 3-like	53024	4.3
syntaxin-1A-like	64986	3.8
dna ligase 1-like	158921	3.8
synaptobrevin-like	63664	3.7
anaphase-promoting complex subunit 2-like	201262	3.5
structural maintenance of chromosomes protein 5-like	62641	3.2
endoribonuclease dicer-like	203025	3.2
protein aatf-like	262855	3.0
CDK-activating kinase assembly factor MAT1-like	62887	3.0
serine threonine-protein kinase nek4	198815	3.0
citron rho-interacting kinase-like	201696	2.8
structural maintenance of chromosomes protein 4-like	51790	2.8
regulator of chromosome condensation-like	50946	2.5
Serine/threonine-protein kinase Chk2-like	157318	2.3
nuclear migration protein nudc-like	210877	2.3
mini-chromosome maintenance complex-binding protein	64008	2.3
structural maintenance of chromosomes protein 2-like	200814	2.2
wee1-like protein kinase-like	264255	2.2
GTP-binding protein	161278	2.1
cyclin-dependent kinase 11-like	260921	2.1
condensin-2 complex subunit d3-like	50977	10.8
SAM domain-containing protein	52335	6.2
SH3 domain-containing protein	53175	6.2
transforming growth factor beta-2 isoform 1	63568	5.1
serine/threonine-protein kinase nek8	156456	4.2
cell division protein FtsH	53814	4.2
mau2 chromatid cohesion factor homolog	52895	3.7
cell division cycle protein 20 homolog	62534	3.7
abnormal spindle-like microcephaly-associated protein homolog	51974	3.5
condensin complex subunit 3	63122	3.2
zinc finger fyve domain-containing protein 26	52835	3.1
ras subfamily protein	261504	2.4
achain structure of the mutant fibroblast growth factor receptor 1	50994	2.3
breast cancer anti-estrogen resistance protein 1	202107	2.1
protein elys-like	261324	2.1

RESPONSE TO STRESS DATA (From Figure 8B)

Most significant BLAST result	Contig ID	Reinf/Apo
hypoxia up-regulated protein 1-like	263453	3.9
transposase domain-containing protein-like	52348	3.8
translation initiation factor eif-2b subunit epsilon-like	59955	3.6
mitochondrial import receptor subunit tom70	201868	2.6
dual specificity protein kinase shkc-like	261231	2.5
metalloendopeptidase oma1, mitochondrial-like	214499	2.1
heat shock 70 kDa protein 12a-like	63971	10.0
s-adenosyl-l-homocysteine hydrolase	53504	7.7
misshapen-like kinase 1-like	51365	3.5
mitogen-activated protein kinase kinase kinase 4-llike	51885	2.6
heat shock protein ibpa	71615	2.4
serine threonine-protein kinase smg1	51829	2.3
eukaryotic translation initiation factor 3 subunit l-like	270473	2.3
mitogen-activated protein kinase kinase kinase mlt-like	52945	2.1
