# Supplementary material for: Transcriptomic analysis of differential host gene expression upon uptake of symbionts: a case study with Symbiodinium and the major bioeroding sponge Cliona varians
Source: BMC Genomics. 2014 May 16;15(1):376. doi: 10.1186/1471-2164-15-376 (PMC4144087; doi:10.1186/1471-2164-15-376)
Supplement: Supplementary file 7 — Additional file 7: Table S3: Comparison of expression levels found in the RNA-Seq experiment for each of the clones pulled out of the Suppressive Subtractive Hybridization library. SSH clone names are provided as are contigs that align to that sequence. The expression levels recorded from the transcriptome are indicated as are the fold differences. Numbers greater than one indicate that the “reinfected” expression is higher than the “aposymbiotic” expression. Numbers less than one indicate that the “aposymbiotic” expression is higher than the “reinfected” expression. (PDF 51 KB) [file 12864_2013_6178_MOESM7_ESM.pdf]

SUPPLEMENTAL TABLE 3

CONCORDANT EXPRESSION PATTERNS OBSERVED IN RNA-Seq & SSH DATASETS

SSH_gene	Plasmid	Contig ID	Aposymbiotic	Reinfected	Fold Difference
3-hydroxybutyrate dehydrogenase type 2	P32C6	271095	63	127.7	2.03
Actin-related protein 2/3 complex subunit	P23C11	243793	128	187.1	2.03
		172567	4	15.2	2.03
ATPase, H+ transporting, lysosomal, V0 subunit	P13C41	53216	3	7.6	2.53
Ca2+-triggered coelenterazine-binding protein 2	P23C14	210676	54	83.4	1.54
Calcium-binding protein p22; Calcineurin	P2C7	62130	51	88.5	1.74
CHK1 checkpoint-like protein	P27C3	171592	12	19	1.58
Creatine kinase U-type, mitochondrial	P32C3	198511	181	228.9	1.26
Cyclophilin A	P16C18	167197	118	158	1.34
		110747	0	1.3	1.30
Ephrin type-B receptor 1; Protein tyrosine kinase	P32C7	274042	2	12.6	1.74
Ferritin	P11C33	195181	26	139.1	5.35
		172589	17	64.5	3.79
		167071	34	92.3	2.71
	P24C13	244173	158	173.2	1.10
	P36C7	244173	158	173.2	1.10
Ficolin-2	P10C21	219184	0	502	502.00
G-protein gamma subunit	P35C2	243780	232	256.7	1.11
Gamma-interferon-inducible lysosomal thiol reductase-like	P34C1	106065	94	279.4	2.97
Glutamine synthetase	P	211494	82	127.7	1.56
Heat shock protein 70	P8C27	194534	43	102.4	2.38
Inorganic pyrophosphatase	P35C14	210752	13	22.8	1.75
		172365	1	3.8	3.80
MafB chain A	P22C7	161183	1073	1165.8	1.09
Neurogenic locus notch protein homolog (neuregulin)	P24C3	271685	39	107.5	2.76
Nuclear pore complex Nup50 (nucleoporin)	P4C11	262759	30	46.8	1.56
Proteasome subunit alpha	P15C16	62102	51	160.6	3.15
Proteasome subunit beta	P1C1	176676	4	26.6	6.65
Ribonuclease K-like; Salivary secreted ribonuclease	P8C8	211219	133	166.9	1.25
		211658	141	244	1.73
Ribosomal proteins	P2C6	217593	39	65.7	1.68
		169342	5	24	4.80
	P8C6	173368	58	112.5	1.94
		172754	28	68.3	2.44
	P8C4	176954	0	1.3	1.30
	P11C27	238142	8	22.8	2.85
	P15C13	172015	176	244	1.39
	P15C15	198299	150	206.1	1.37
	P31C6	173368	58	112.5	1.94
		172754	28	68.3	2.44
RNA polymerase-associated protein LEO1	P11C31	62675	17	31.6	1.86
Selenoprotein Jb; J1a crystallin	P24C6	106592	177	209.9	1.19
Serum response factor	P27C2	196572	839	921.7	1.10
	P35C12	196572	839	921.7	1.10
Sulfide quinone reductase	P34C7	201177	119	292.1	2.45
Thymosin beta	P23C16	219534	494	584.1	1.18
Tubulin alpha chain	P9C12	168747	63	150.5	2.39
Vacuolar sorting protein; sortilin-related receptor	P14C3	159535	0	17.7	17.70
WAS protein family homolog 1 (DAF)	P14C7	211902	15	65.7	4.38
Hypothetical proteins	P14C8	167347	13	127.7	9.82
		117374	1	1.3	1.30
	P16C17	167347	13	127.7	9.82
		117374	1	1.3	1.30

CONFLICTING EXPRESSION PATTERNS OBSERVED IN RNA-Seq & SSH DATASETS

SSH_gene	Plasmid	Contig ID	Aposymbiotic	Reinfected	Fold Difference
AP-2 complex subunit beta (adaptor-related protein)	P23C6	245152	22	13.9	0.63
		279831	1	2.5	2.50
Cytoskeletal actin	P8C5	202762	21	35.4	1.69
		204121	869	441.3	0.51
		225184	528	288.3	0.55
	P11C32	202762	21	35.4	1.69
		204121	869	441.3	0.51
		225184	528	288.3	0.55
Deleted in malignant brain tumors 1 protein-like;Scavenger receptor cysteine-rich type protein	P11C30	262647	336	98.6	0.29
		195761	113	163.1	1.44
	P23C4	262647	336	98.6	0.29
		195761	113	163.1	1.44
Dihydrolipoyl dehydrogenase	P16C20	167771	3	40.5	13.50
		162396	6	0	0.17
		213269	9	20.2	2.24
Dynein heavy chain	P20C8	262328	402	193.5	0.48
		7062	0	1.3	1.30
Tyrosine 3-monooxygenase/tryptophan 5-monooxygenase activation protein (YWHAE) (14-3-3 superfamily)	P25C4	172006	55	87.2	1.59
		169709	59	39.2	0.66
von Willebrand factor A domain-containing protein-5a	P10C23	206365	9	6.3	0.70
		156990	12	12.6	1.05
		157297	8	7.6	0.95

NB: BLAST searches of the transcriptome using cyplasin, two hypothetical proteins, and two ribosomal sequences did not retrieve significant alignments. Thus we were unable to determine the direction of expression values for these five genes.
